# Synthesis, Characterization and Molecular Docking of Novel Bioactive Thiazolyl-Thiazole Derivatives as Promising Cytotoxic Antitumor Drug

**DOI:** 10.3390/molecules21010003

**Published:** 2015-12-22

**Authors:** Sobhi M. Gomha, Taher A. Salaheldin, Huwaida M. E. Hassaneen, Hassan M. Abdel-Aziz, Mohammed A. Khedr

**Affiliations:** 1 Department of Chemistry, Faculty of Science, Cairo University, Giza 12613, Egypt; huwaida30@gmail.com; 2 Nanotechnology and Advanced Materials Central Lab, Agricultural Research Center, Giza 12613, Egypt; t1salah@hotmail.com; 3 Chemistry Department, Faculty of Science, Cairo University, Bani Suef Branch, Bani Suef 62514, Egypt; dr_hassan1971@yahoo.com; 4 Department of Pharmaceutical Chemistry, Faculty of Pharmacy, Helwan University, Ein Helwan, Cairo 11795, Egypt; mohammed_abdou0@yahoo.com; 5 Department of Pharmaceutical Sciences, College of Clinical Pharmacy, King Faisal University, P. O. 380, Al-Hasaa 31982, Saudi Arabia

**Keywords:** ethylidenethiocarbohydrazide, ethylidenethiosemicarbazide, hydrazonoyl halides, 1,3-thiazole, 1,3,4-thiadiazole, cytotoxic activity, molecular docking

## Abstract

Reactions of ethylidenethiocarbohydrazide with hydrazonoyl halides gave 1,3-thiazole or 1,3,4-thiadiazole derivatives according to the type of hydrazonoyl halides. Treatment of ethylidenethiosemicarbazide with hydrazonoyl halides and dimethylacetylene dicarboxylate (DMAD) afforded the corresponding arylazothiazoles and 1,3-thiazolidin-4-one derivatives, respectively. The structures of the synthesized products were confirmed by IR, ^1^H-NMR, ^13^C-NMR and mass spectral techniques. The cytotoxic activity of the selected products against the Hepatic carcinoma cell line (Hepg-2) was determined by MTT assay indicating a concentration dependent cellular growth inhibitory effect, especially for compounds **14c** and **14e**. The dose response curves indicated the IC_50_ (the concentration of test compounds required to kill 50% of cell population) were 0.54 μM and 0.50 μM, respectively. Confocal laser scanning imaging of the treated cells stained by Rhodamin 123 and Acridine orange dyes confirmed that the selected compounds inhibit the mitochondrial lactate dehydrogenase enzymes. The binding mode of the active compounds was interpreted by a molecular docking study. The obtained results revealed promising cytotoxic activity.

## 1. Introduction

Hepatocellular Carcinoma (HCC) is considered the fifth most common cancer type worldwide and the third most common cause of cancer mortality. Globally, over 560,000 people develop liver cancer each year and an almost equal number, 550,000, die of it [1].

At stage I, surgical resection or transplantation is considered a potentially curative modality for HCC; patients with localized unrespectable disease are usually treated with some form of localized therapy. Local therapeutic modalities include targeted chemotherapy through hepatic artery combined with embolization, percutaneous ethanol ablation, radio embolization, radiofrequency ablation, and cryosurgery [2]. Thus, novel approaches for the treatment of unrespectable advanced or metastatic HCC represents a high-unmet medical need. In this work, novel thiazole derivatives were used as a module for management of HCC. Thiazoles are a familiar group of heterocyclic compounds possessing a wide variety of biological activities, and their usefulness as medicines are well established. Thiazole derivatives are reported to exhibit diverse biological activities as antimicrobial [3,4,5], antioxidant [6], antitubercular [7], and anticonvulsant [8], and anticonvulsant, anticancer [9,10] agents. Moreover, many derivatives of thiazoles are used as selective Cyclooxygenase-2 Inhibitors [11], in addition to their use as 5-HT3 receptor antagonists [12] and as potent and selective acetyl Co-A carboxylase-2 inhibitors [13]. Furthermore, the interesting properties of thiazole derivatives [14,15] in relation to the various changes in the structures of these compounds is worth studying for the synthesis of some less toxic and more potent drugs. Thus, the introduction of other heterocyclic moieties (as the thiazole and thiadiazole ring) should certainly help to fulfill this objective. The aforementioned biological, pharmacological, and industrial importance of these derivatives prompted our interest for the synthesis of some new examples of this class of compounds.

As a part of our research interest towards developing new routes for the synthesis of a variety of heterocyclic systems with promising antitumor activities [9,10,16,17,18,19,20,21,22], we report in the present work the synthesis of a new series of thiazolyl-thiazoles as promising cytotoxic antitumor drugs.

## 2. Results and Discussion

### 2.1. Chemistry

**Scheme 1:**
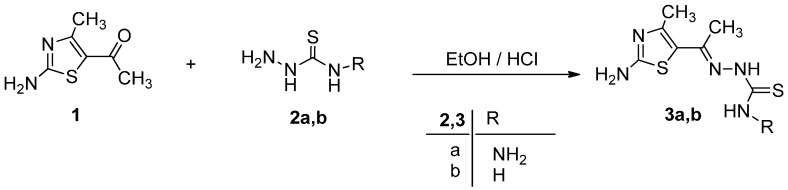
Synthesis of ethylidenethiocarbohydrazide derivative **3a** and ethylidenethiosemicarbazide derivative **3b**.

Ethylidenethiocarbohydrazide (**3a**) and ethylidenethiosemicarbazide (**3b**) were prepared via condensation of 5-acetyl-2-amino-4-methylthiazole (**1**) [23] with thiocarbohydrazide (**2a**) and thiosemicarbazide (**2b**) in absolute ethanol in the presence of a catalytic amount of concentrated HCl as depicted in Scheme 1.

The structure elucidation of the products **3a** and **3b** were substantiated through spectral data. The mass spectrum of isolated product (*m/z* 245) was consistent with the expected product **3a**. The ^1^H-NMR spectrum of **3a** showed broad singlet bands for NH_2_ and NH groups of the thiocarbohydrazide moiety at 4.92, 9.62 and 14.12 ppm, respectively, and the band for NH_2_ group of the thiazole ring at 7.42 ppm as well as the two singlet bands for methyl groups at 2.42 and 2.85 ppm.

**Scheme 2:**
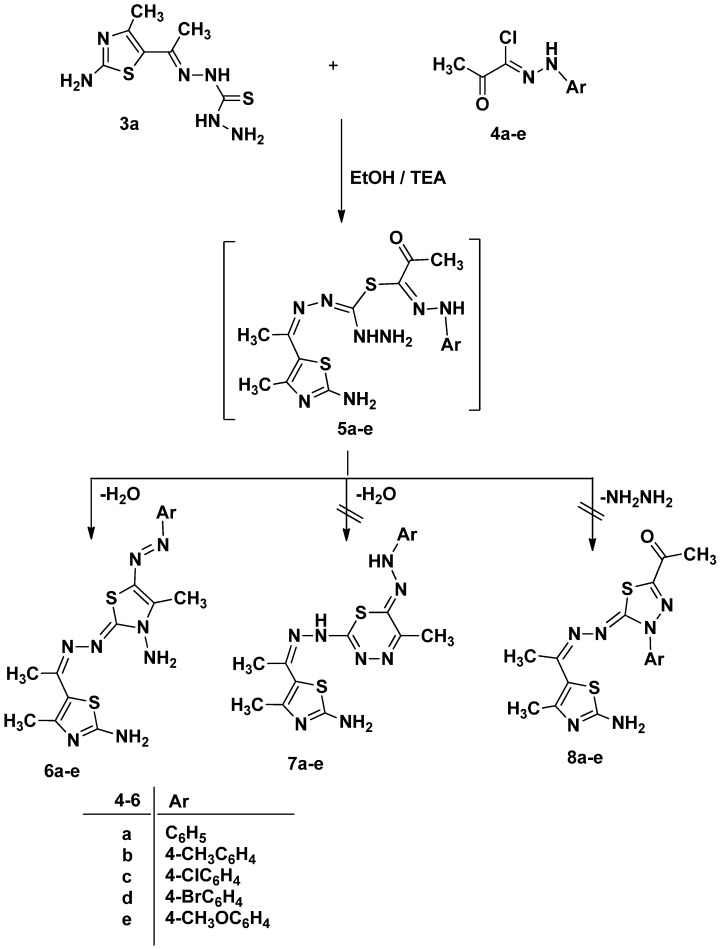
Synthesis of 1,3-thiazole derivatives **6a**–**e**.

We commenced our study on the reactivity of ethylidenethiocarbohydrazide **3a** toward different types of hydrazonoyl halides **4a**–**e** and **9** to investigate the effect of the presence of carbonyl group on the course of the reaction. Initially, ethylidenethiocarbohydrazide **3a** reacted with α-keto-hydrazonoyl halide **4a**–**e** in refluxing ethanol in presence of triethylamine to give dark red color products that proved we are isolated azo-thiazole derivatives **6a**–**e** and not the hydrazo-thiadiazine derivatives **7a**–**e** via elimination of hydrochloric acid and water as shown in Scheme 2. Spectroscopic analyses (IR, MS, ^1^H- and ^13^C-NMR) confirmed the structure of 1,3-thiazole derivatives **6a**–**e** and not **7a**–**e** was isolated. The ^1^H-NMR spectrum showed two broad singlet signal for two NH_2_ groups at ~2.5 and ~7.11 ppm.

**Scheme 3:**
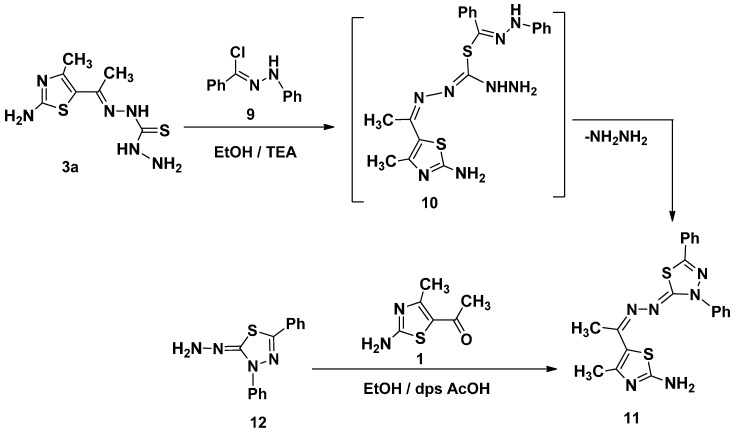
Synthesis of 1,3,4-thiadiazole **11**.

The reaction of *N*′-phenylbenzohydrazonoyl chloride **9** not containing α-haloketone with **3a** in the same condition gave only one isolable product as examined by thin layer chromatography (TLC) The molecular formula of **11**, C_20_H_18_N_6_S_2_, was consistent with elimination of HCl and NH_2_NH_2_. The structure of **11** was elucidated by elemental analysis and spectroscopic data (see Experimental Section) (Scheme 3). Compound **11** was also obtained via the reaction of 5-acetyl-2-amino-4-methylthiazole (**1**) with 2-hydrazono-3,5-diphenyl-2,3-dihydro-1,3,4-thiadiazole (**12**) [24] in ethanol in presence of drops of acetic acid afford product identical in all respects with that obtained from reaction of the **9** with of ethylidenethiocarbohydrazide **3a** (Scheme 3).

**Scheme 4:**
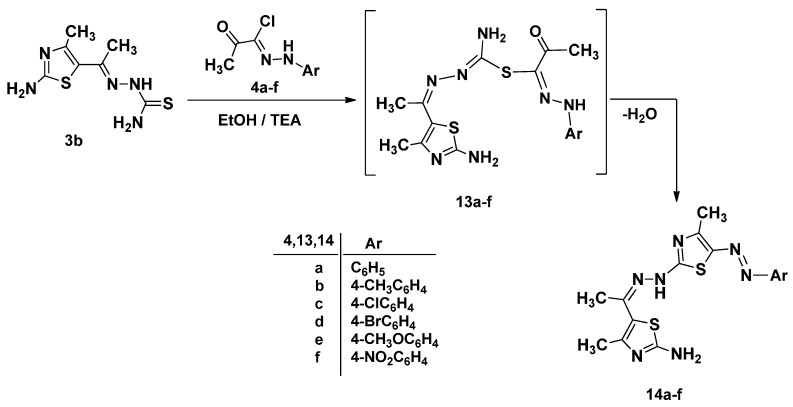
Synthesis of 5-hydrazono-thiazole derivatives **14a**–**f**.

Next, the reaction of 1-[1-(2-amino-4-methylthiazol-5-yl)ethylidene]thiosemicarbazide **3b** with α-keto-hydrazonoyl halides **4a**–**f** was performed under similar reaction conditions, and afforded the final product 5-hydrazono-thiazole derivatives **14a**–**f** via same route of elimination HCl and H_2_O (Scheme 4) [25]. The ^1^H-NMR of **14a** exhibited three singlet signals for methyl groups and a multiplet resonance at δ 6.95–7.35 for aromatic group (see Experimental Section & Figure S3).

**Scheme 5:**
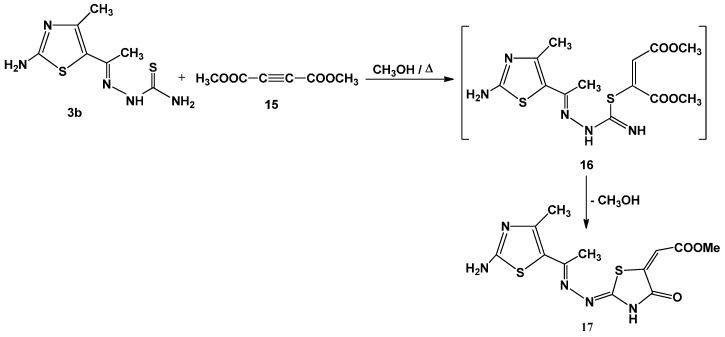
Synthesis of thiazolidin-4-one **17**.

Finally, refluxing an equimolecular mixture of **3b** and dimethylacetylenedicarboxylate **15** in methanol yielded methyl 4-oxo-thiazolidin-5-ylidene acetate **17** (Scheme 5). The structure of **17** was established on the basis of analytical and spectral data. Thus, the ^1^H-NMR spectrum showed singlet signal at δ 12.77 ppm (D_2_O-exchangeable), assignable to NH group. In addition, the presence of a signal at δ 6.60 assigned to the =C*H*, and singlet at δ 3.29 for ester methyl protons [9,26,27,28].

### 2.2. Cytotoxiclogical Activity against HEP G2 Cell Line

**Figure 1:**
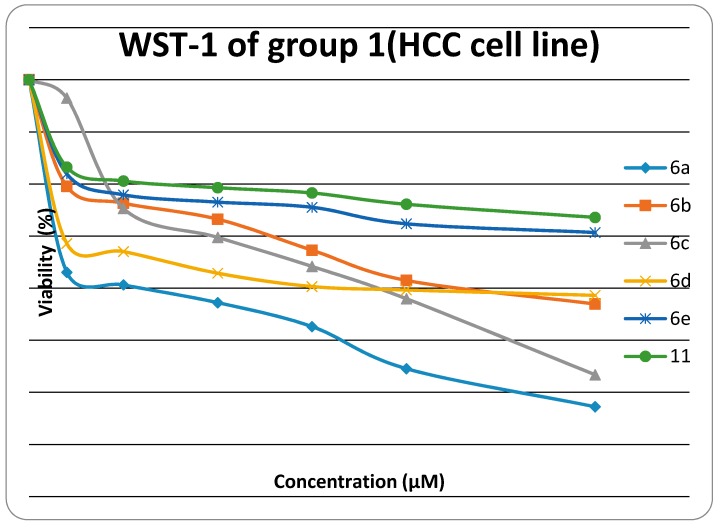
Viability chart of tested Group 1 compounds against HEP G2 cell line.

**Figure 2:**
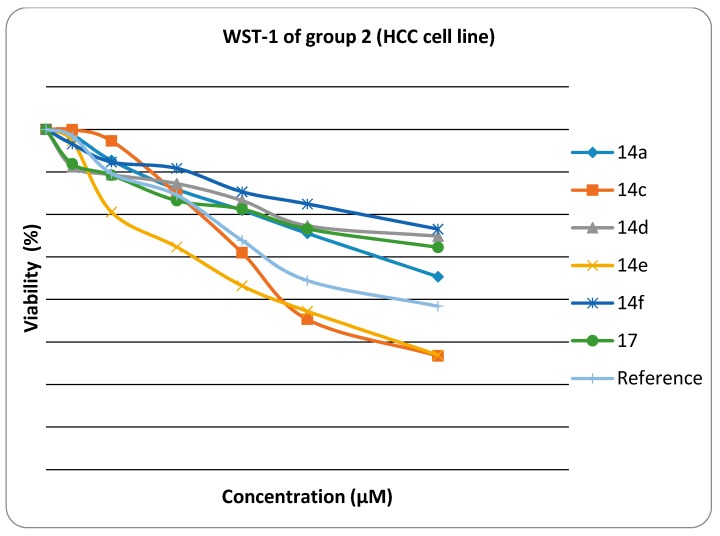
Viability chart of tested Group 2 compounds against HEP G2 cell line.

The cytotoxiclogical activity of synthesized products Group 1 (**6a**, **6b**, **6c**, **6d**, **6e**, **6f**, **11**) and Group 2 (**14a**, **14c**, **14d**, **14e**, **14f**, **17**) were evaluated against HEP G2) using the WST-1 cell proliferation assay as a fast and sensitive quantification of cell proliferation and viability. In brief, the assay is based on the cleavage of the tetrazolium salt WST-1 to formazan by cellular mitochondrial dehydrogenases. Expansion in the number of viable cells results in an increase in the overall activity of the mitochondrial dehydrogenases in the sample. The augmentation in enzyme activity leads to the increase in the amount of formazan dye formed. The formazan dye produced by viable cells can be quantified by a multiwell spectrophotometer (microplate reader) by measuring the absorbance of the dye solution at 450 nm. The viability of the tested compounds in response to their concentration is illustrated in Figure 1 and Figure 2. Data generated were used to plot a dose response curve to determine the concentration of test compounds required to kill 50% of cell population (IC_50_) was estimated exponentially.

Cytotoxic activity was expressed as the mean IC_50_ of three independent experiments. The results are represented in Table 1.

**Table 1:** IC_50_ values of tested compounds ± standard deviation against HEP G2.

Compound No.	IC_50_ (μM)	Compound No.	IC_50_ (μM)
Doxorubicin	0.68 ± 0.03	**14a**	0.84 ± 0.04
**6a**	1.00 ± 0.08	**14c**	0.52 ± 0.03
**6b**	1.49 ± 0.1	**14d**	1.19 ± 0.09
**6c**	1.04 ± 0.07	**14e**	0.50 ± 0.02
**6d**	1.73 ± 0.12	**14f**	1.28 ± 0.08
**6e**	2.17 ± 0.13	**17**	1.07 ± 0.06
**11**	2.91 ± 0.15		

The results presented in Table 1 showed that:

The *in vitro* inhibitory activities of tested compounds against the hepatocellular carcinoma cell line (HEP G2) have the descending order as follow: **14e** > **14c** > **14a** > **6a** > **6c** > **17** >**14d** >**14f** > **6b** > **6d** > **6e** > **11**. The thiazole rings **6a**–**e** and **14a**–**f** have better *in vitro* inhibitory activity than the thiadiazole ring **11**. The thiazolone ring **17** has better *in vitro* inhibitory activity than the thiadiazole ring **11**. The introduction of a amino group on N3 of thiazole ring increases the activity when fixing the substituent at 4-position of phenyl group at position 5 in the thiazole ring (thiazole **14a** is more active than thiazole **6a,** thiazole **14c** is more active than thiazole **6c**, thiazole **14d** is more active than thiazole **6d**, and thiazole **14e** is more active than thiazole **6e**).

The results revealed that compounds **14e**, **14c** and **14a** (IC_50_ were 0.5 ± 0.02 µM, 0.52 ± 0.03 µM and 0.84 ± 0.04 µM, respectively) have promising antitumor activity against hepatocellular carcinoma cell line (HEP G2) when compared to doxorubicin as a reference drug (IC_50_ value of doxorubicin = 0.68 ± 0.03 µM), while **6a**, **6c**, **17**, **14d** and **14f** have moderate activity (IC_50_ were 1.04 ± 0.07 µM, 1.07 ± 0.06 µM, 1.19 ± 0.09 µM, and 1.28 ± 0.08 µM, respectively). On the other hand, **6b**, **6d**, **6e** and **11** have lower inhibitory activity against (HEP G2) (IC_50_ = 1.49 ± 0.1 µM, 1.73 ± 0.12 µM, 2.17 ± 0.13 µM and 2.91 ± 0.15 µM, respectively). The small values of IC_50_ for the selected compounds indicate that, for more anticancer effect, higher concentrations can be used.

**Figure 3:**
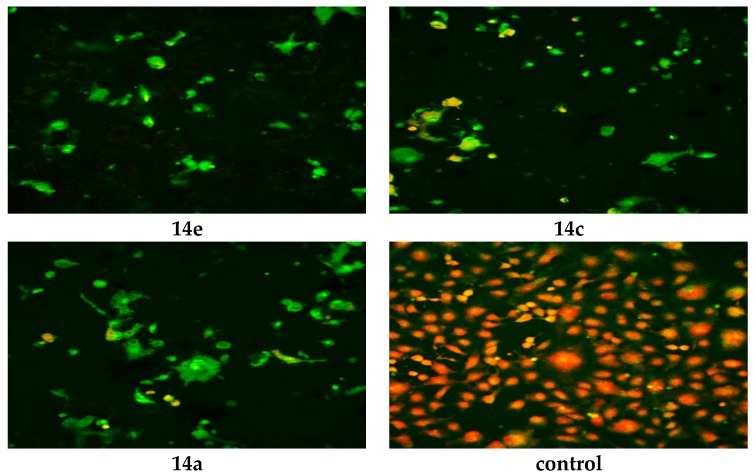
Confocal Laser Scanning Microscopy (CLSM) image of HEP G2 cell line treated with 0.6 μM tested compounds (**14e**, **14c**, **14a** and untreated control). Stained by Acridine orange (green) and Rodamine 123 (Orange).

**Figure 4:**
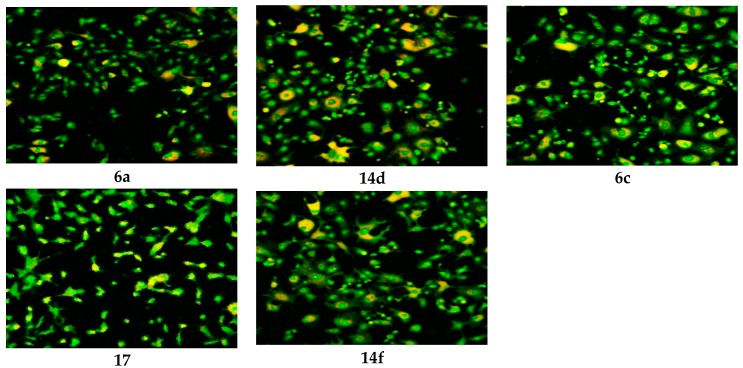
CLSM image of HEP G2 cell line treated with 0.6 μM tested compounds (**6a**, **6c**, **17**, **14d** and **14f**). Stained by Acridine orange (green) and Rodamine 123 (Orange).

**Figure 5:**
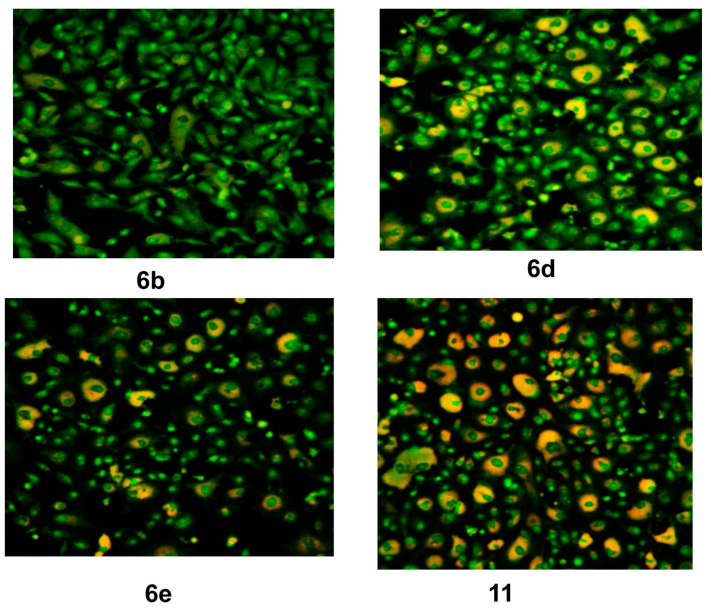
CLSM image of HEP G2 cell line treated with 0.6 μM tested compounds (**6b**, **6d**, **6e** and **11**). Stained by Acridine orange (green) and Rodamine 123 (Orange).

The WST-1 assay results revealed a significant decrease in the mitochondrial dehydrogenase activity as a function of the growth rate of the tumor cells but did not explain the mode of action of the compounds. Confocal laser scanning microscopic (CLSM) imaging of HEP G2 cell line stained with acridine orange dye for nucleic acids (green stain) and Rodamine 123 (Orange stain) for inner mitochondrial membrane, where the dehydrogenases located, reflects the activity of mitochondrial dehydrogenase enzyme activity. It was obvious that the activity of dehydrogenases significantly decreased with cells treated with **14e**, **14c** and **14a** compared to the untreated control (Figure 3), while moderate decrease for **6a**, **6c**, **17**, **14d** and **14f** (Figure 4), and mild decrease for the rest of the compounds (Figure 5). The higher amount of orange color reflects the higher activity of mitochondrial dehydrogenase enzyme, which means higher viability and *vice versa*.

### 2.3. Molecular Docking Study

**Figure 6:**
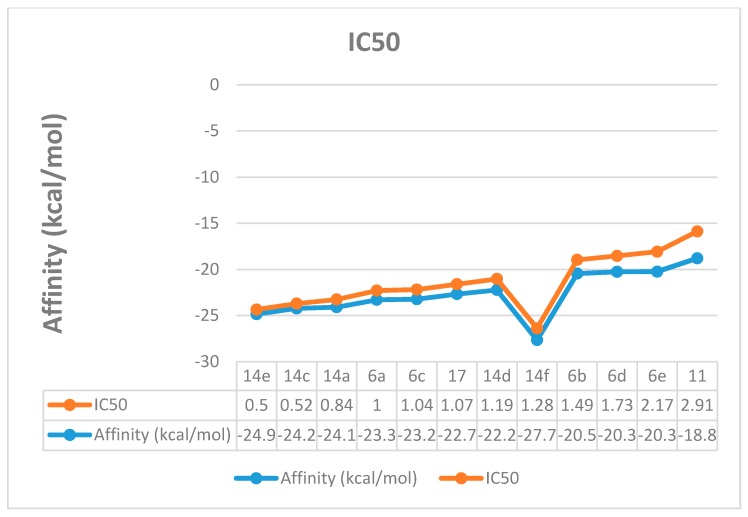
Correlation between the docking affinity and the IC_50_.

In cancer cells, the increased glucose uptake results in high glycolytic activity, which, in turn, causes elevated levels of lactate production [29]. The lactate production is controlled by lactate dehydrogenase-5 (LDH-5), an isoenzyme from the lactate dehydrogenase that is mainly found in the liver cells [30]. It has been reported that LDH-5 has an important role in tumor maintenance in many human cancers like the hepatic cancer [31]. Recently, LDH-5 inhibitors have been reported to show potential anticancer activity [32], which confirms that LDH-5 inhibition is a good target for developing anticancer agents. Molecular docking study was conducted to interpret the LDH inhibitory activity for the synthesized compounds that were confirmed biologically. The docking was performed using Leadit 2.1.5 software to calculate and analyze all parameters that may have a direct relationship to the LDH inhibition (Table 2). A direct correlation between the activity and the binding affinity was observed (Figure 6). For example, compound **14e** showed the best affinity (–24.85 kcal/mol) and the best IC_50_ as well. The entropy ligand conformation score for the highly active group was a neglected value (0.00), which is favored for best fitting.

There is no doubt that all the tested compounds showed promising cytotoxic activity against HEP G2 cell line in low micromolar range ranged from 0.50 μM (compound **14e**) to 2.91 μM (compound **11**). All compounds shared the same 5-(substituted)-ethylidene-hydrazono-2-amine-4-methyl-1,3-thiazole scaffold. This scaffold showed the ability to form some interactions with the main residues in the active site of LDH-5 that was clear in the NH-N= moiety hydrogen bond formation with Arg 99, and the 2-amino group of the 1,3-thizole ring and its hydrogen bond formation with Gly 29, Gly 27, and Thr 95. The compounds were divided into three groups according to their activity: highly active group (**14e**, **14c**, and **14f**), moderately active group (**6a**, **6c**, **17**, **14d**, and **14f**) and low active group (**6b**, **6d**, **6e** and **11**).

**Table 2:** Docking Results of the active compounds using Leadit 2.1.5 software (software license was purchased from BioSolveIT GmbH, Germany).

Compounds	Affinity Score kcal/mol	Lipophilic Contribution Score	Clash Score	Ligand Entropy Conformation Score
**14e**	−24.85	−8.50	4.32	0.00
**14c**	−24.23	−8.56	4.54	0.00
**14a**	−24.10	−12.27	4.12	0.00
**6a**	−23.50	−8.31	5.82	1.40
**6c**	−23.23	−8.28	5.88	1.40
**17**	−22.68	−6.36	3.85	1.40
**14d**	−22.23	−13.86	7.55	0.00
**14f**	−21.66	−6.91	8.23	0.00
**6b**	−20.46	−8.52	5.89	1.40
**6d**	−20.27	−8.31	5.88	1.40
**6e**	−20.25	−8.39	4.54	2.80
**11**	−18.80	−9.23	5.11	0.00

**Figure 7:**
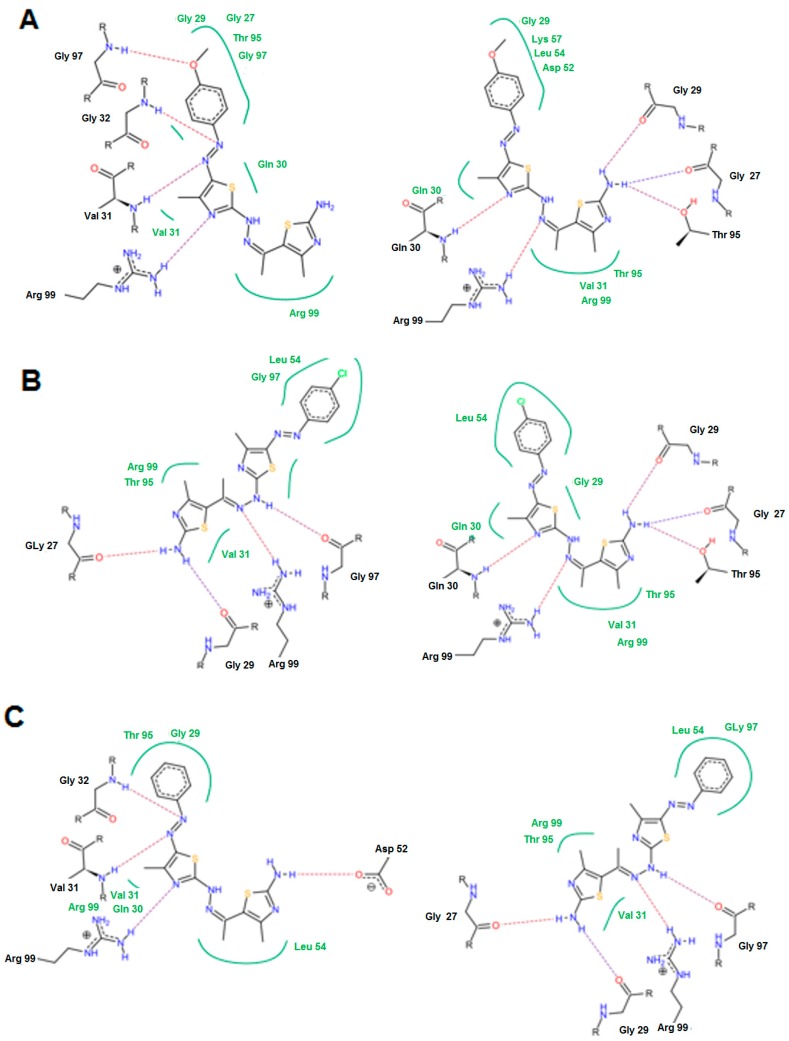
Possible binding modes of compounds (**A**) **14e**; (**B**) **14c**; and (**C**) **14a**.

For the highly active group, a common binding mode was observed in which the Gly 97, Gly 32, Val 31, Arg 99, Gln 30 and Thr 95 were the most common involved residues. The N=N, C=N, NH_2_, and OCH_3_ groups were the most important groups for formation of the hydrogen bonds (Figure 7). The oxygen atom of the OCH_3_ group in case of **14e** interacted with Gly 97 to form a hydrogen bond. This feature was absent in compound **14c** with the *p*-chloro phenyl and compound **14a** with the non-substituted phenyl ring.

**Figure 8:**
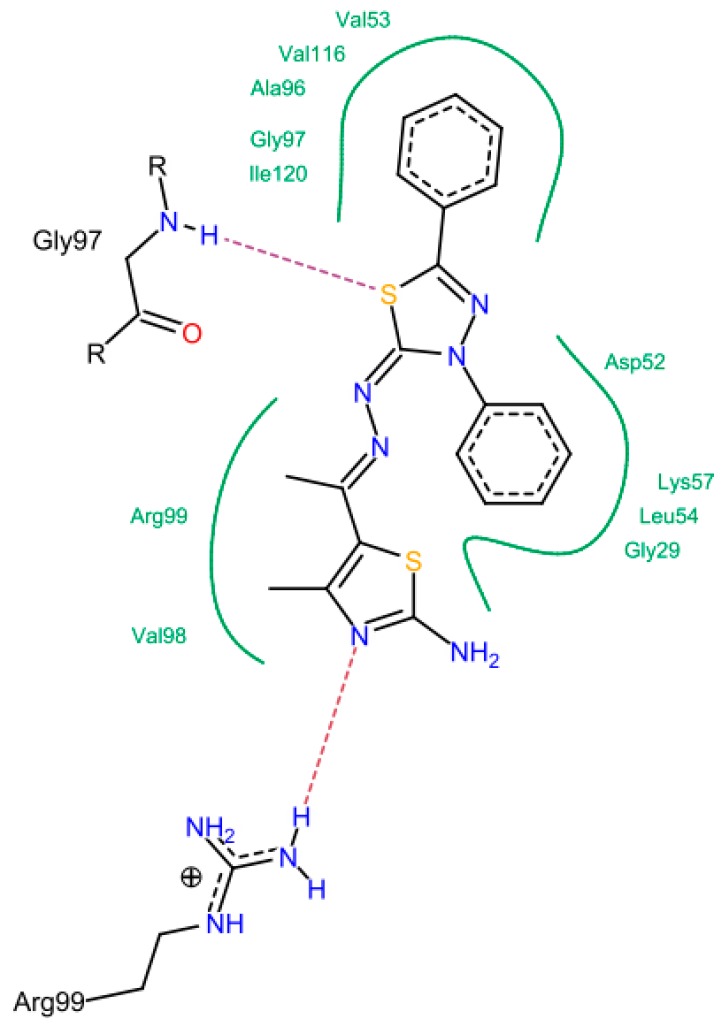
The possible binding mode of compound **11**.

In the moderately active group (**6a**, **6c**, **17**, **14d**, and **14f**), the presence of 3-amino group in the thiazol-2-(3*H*) moiety was important to form a hydrogen bond with Thr 99, Asn 113 and Ala 96. The N=N and C=N groups were involved in the interactions. An overall good fitting and placement was observed for the docked compounds inside the active site where the nicotinamide-adenine dinucleotide was bound. Regarding the low active group (**6b**, **6d**, **6e**, and **11**)**,** the most obvious result was in compound **11** that has a 3-phenyl thiadiazole moiety. This phenyl ring restricts the interactions and the flexibility of the compound resulting in a low binding affinity. The steric hindrance of compound **11** resulted from the 3,5-diphenyl-2,3-dihydro-1,3,4-thiadiazole ring system that was the main reason for bad fitting and low affinity (Figure 8).

Generally, in the binding site area with mild hydrophobic amino acids, like Ala 96, Gly 97 and Gly 32, the activity was achieved by non-substituted phenyl ring, -OCH_3_ substitution, and/or chloro substitution that do not affect strongly the electron cloud on the phenyl ring. The main interactions in this area are not hydrophobic but, hydrogen bonding so, any substitution by bromo atom or nitro group affected the electron cloud and the π-π system resulting in shifting in the activity.

## 3. Experimental Section

### 3.1. Chemistry

Melting points were measured on an Electrothermal IA 9000 series digital melting point apparatus (Bibby Sci. Lim. Stone, Staffordshire, UK). IR spectra were recorded in potassium bromide discs on Shimadzu FTIR 8101 PC infrared spectrophotometer (Shimadzu, Tokyo, Japan). NMR spectra were recorded on a Varian Mercury VX-300 NMR spectrometer (Varian, Inc., Karlsruhe, Germany) operating at 300 MHz (^1^H-NMR) or 75 MHz (^13^C-NMR) and run in deuterated dimethylsulfoxide (DMSO-*d*_6_). Chemical shifts were related to that of the solvent. Mass spectra were recorded on a Shimadzu GCMS-QP1000 EX mass spectrometer at 70 eV (Tokyo, Japan). Elemental analyzes were measured by using a German made Elementar vario LIII CHNS analyzer (GmbH & Co. KG, Hanau, Germany). Antitumor activity was evaluated by the Nanotechnology & Advanced materials central lab, Agricultural Research Center, Giza, Egypt. Hydrazonoyl halides were prepared as previously reported in the respective literature [33,34].

#### 3.1.1. Synthesis of **3a** and **3b**

5-Acetyl-2-amino-4-methylthiazole **1** (7.8 g, 50 mmol) was dissolved in 100 mL of absolute ethanol and stirred with an equimolar quantity of thiocarbohydrazide (**2a**) or thiosemicarbazide (**2b**) for 24 h at room temperature with catalytic amounts of concentrated HCl. The desired products precipitated from reaction mixture were filtered, washed with ethanol and recrystallized from acetic acid to give pure product of compound **3a** and **3b**.

*1-[1-(2-Amino-4-methylthiazol-5-yl)ethylidene]thiocarbohydrazide* (**3a**). White crystals (84%); mp 234–236 °C; IR (KBr): *v* 3436, 3231 (NH_2_), 3182 (NH), 1631(C=N), 1258 (C=S) cm^−1^; ^1^H-NMR (DMSO-*d*_6_): δ 2.42 (s, 3H, CH_3_), 2.85(s, 3H, CH_3_-thiazole), 4.92 (s, br, 2H, D_2_O-exchangeable, NH_2_, CSNHNH_2_), 7.42 (s, br, 2H, D_2_O-exchangeable, NH_2_-thiazole), 9.64 (s, br, 1H, D_2_O-exchangeable, NH, CSNHNH2), 14.12 (s, br, 1H, D_2_O-exchangeable, NH, C=N-NH-CS); MS *m/z* (%): 245 (M^+^, 19), 244 (M^+^, 21), 219 (27), 122 (29), 79 (100). Anal. Calcd. for C_7_H_12_N_6_S_2_ (244.34): C, 34.41; H, 4.95; N, 34.39. Found C, 34.48; H, 4.86; N, 34.24%.

*1-[1-(2-Amino-4-methylthiazol-5-yl)ethylidene]thiosemicarbazide* (**3b**)**.** White crystals (76%); mp 245–247 °C; IR (KBr): ν 3406, 3275 (NH_2_), 3217 (NH), 1639 (C=N), 1242 (C=S) cm^−1^; ^1^H-NMR (DMSO-*d*_6_): δ 2.42 (s, 3H, CH_3_), 2.86 (s, 3H, CH_3_-thiazole), 4.08 (s, br, D_2_O-exchangeable, 2H, NH_2_), 7.45(s, br, 2H, D_2_O-exchangeable, NH_2_-thiazole), 9.78 (s, br, 1H, D_2_O-exchangeable, NH); MS *m/z* (%): 229 (M^+^, 6), 212 (7), 128 (19), 42 (100). Anal. Calcd. for C_7_H_11_N_5_S_2_ (229.33): C, 36.66; H, 4.83; N, 30.54. Found C, 36.60; H, 4.69; N, 30.41%.

#### 3.1.2. Synthesis of 5-[1-((3-Amino-4-methyl-5-(substitutedphenyldiazenyl)thiazol-2(3*H*)-ethylidene) hydrazono)]-2-amine-4-methyl-1,3-thiazole (**6a**–**e**)

A mixture of 1-[1-(2-amino-4-methylthiazol-5-yl)ethylidene]thiocarbohydrazide **3a** (0.244 g, 1 mmol) and appropriate hydrazonoyl halides **4a**–**e** (1 mmol) in dioxan (30 mL) containing triethylamine (0.1 g, 1 mmol) was refluxed for 6–8 h (monitored by TLC). The formed precipitate after cooling at room temperature was isolated by filtration, washed with methanol, dried and recrystallized from appropriate solvent to give products **6a**–**e**.

*5-[1-((3-Amino-4-methyl-5-(phenyldiazenyl)thiazol-2(3H)-ethylidene)hydrazono)]-2-amine-4-methyl-1,3-thiazol* (**6a**). Red solid (67%); mp 192–194 °C; IR (KBr): ν 3406, 3298, 3121 (2NH_2_), 1639 (C=N), 1552 (N=N) cm^−1^; ^1^H-NMR (DMSO-*d*_6_): δ 2.42 (s, 3H, CH_3_), 2.52 (s, br., 2H, D_2_O-exchangeable, NH_2_), 2.85 (s, 3H, CH_3_), 3.43 (s, 3H, CH_3_), 7.15(s, br., 2H, D_2_O-exchangeable, NH_2_), 7.31–7.73 (m, 5H, Ar-H); ^13^C-NMR (DMSO-*d*_6_): δ 16.21 (CH_3_), 17.10 (CH_3_), 19.89 (CH_3_), 114.67, 120.32, 121.56, 128.61, 139.54, 143.14 (Ar-C), 154.15, 161.67, 167.45, 169.78, 176.75 (C=N); MS *m/z* (%): 387 (M^+^ + 1, 6), 387 (M^+^, 8), 163 (18), 110 (67), 77 (100). Anal. Calcd. for C_16_H_18_N_8_S_2_ (386.50): C, 49.72; H, 4.69; N, 28.99. Found C, 49.65; H, 4.60; N, 28.78%.

*5-[1-((3-Amino-4-methyl-5-(4-methylphenyldiazenyl)thiazol-2(3H)-ethylidene)hydrazono)]-2-amine-4-methyl-1,3-thiazol* (**6b**). Red solid (68%); mp 216–218°C; IR (KBr): ν 3407, 3317, 3113 (2NH_2_), 1636 (C=N), 1556 (N=N) cm^−1^; ^1^H-NMR (DMSO-*d*_6_): δ 2.23 (s, 3H, CH_3_), 2.42 (s, 3H, CH_3_), 2.50 (s, br., 2H, D_2_O-exchangeable, NH_2_), 2.84(s, 3H, CH_3_), 3.47 (s, 3H, CH_3_), 7.11 (s, br., 2H, D_2_O-exchangeable, NH_2_), 7.32–7.75 (m, 4H, Ar-H); MS *m/z* (%): 402 (M^+^ + 1, 11), 401 (M^+^ + 1, 18), 400 (M^+^, 29), 233 (19), 153 (69), 105 (100). Anal. Calcd. for C_17_H_20_N_8_S_2_ (400.52): C, 50.98; H, 5.03; N, 27.98. Found C, 50.68; H, 5.00; N, 27.69%.

*5-[1-((3-Amino-5-((4-chlorophenyl)diazenyl)-4-methylthiazol-2(3H)-ethylidene)hydrazono)]-2-amine-4-methyl-1,3-thiazole* (**6c**). Red solid (65%); mp 243–245 °C; IR (KBr): ν 3409, 3312, 3151 (2NH_2_), 1637 (C=N), 1553 (N=N) cm^−1^; ^1^H-NMR (DMSO-*d*_6_): δ 2.43 (s, 3H, CH_3_), 2.52 (s, br., 2H, D_2_O-exchangeable, NH_2_), 2.86 (s, 3H, CH_3_), 3.49 (s, 3H, CH_3_), 7.35 (s, br., 2H, D_2_O-exchangeable, NH_2_), 7.38-7.83 (m, 4H, Ar-H); MS *m/z* (%): 422 (M^+^ + 2, 2), 421 (M^+^ + 1, 4), 420 (M^+^, 10), 299 (17), 244 (43), 212 (46), 155 (52), 113 (85), 72 (100). Anal. Calcd. for C_16_H_17_ClN_8_S_2_ (420.94): C, 45.65; H, 4.07; N, 26.62. Found C, 45.60; H, 4.14; N, 26.38%.

*5-[1-((3-Amino-5-(4-bromophenyl)diazenyl)-4-methylthiazol-2(3H)-ethylidene)hydrazono)]-2-amine-4-methyl-1,3-thiazol* (**6d**). Red solid (66%); mp 218–219 °C; IR (KBr): ν 3409, 3294, 3113 (2NH_2_), 1632 (C=N), 1557 (N=N) cm^−1^; ^1^H-NMR (DMSO-*d*_6_): δ 2.46 (s, 3H, CH_3_), 2.51 (s, br., 2H, D_2_O-exchangeable, NH_2_), 2.85 (s, 3H, CH_3_), 3.47 (s, 3H, CH_3_), 7.26 (s, br., 2H, D_2_O-exchangeable, NH_2_), 7.30–7.81 (m, 4H, Ar-H); MS *m/z* (%): 467 (M^+^ + 2, 15), 466 (M^+^ + 1, 19), 465 (M^+^, 21), 405 (14), 299 (19), 153 (86), 113 (62), 65 (100). Anal. Calcd. for C_16_H_17_BrN_8_S_2_ (465.39): C, 41.29; H, 3.68; N, 24.08. Found C, 41.11; H, 3.57; N, 24.01%.

*5-[1-((3-Amino-5-(4-methoxyphenyl)diazenyl)-4-methylthiazol-2(3H)-ethylidene)hydrazono)]-2-amine-4-methyl-1,3-thiazol* (**6e**). Red solid (68%); mp 212–214 °C; IR (KBr): ν 3403, 3298, 3117 (2NH_2_), 1635 (C=N), 1555 (N=N) cm^−1^; ^1^H-NMR (DMSO-*d*_6_): δ 2.43 (s, 3H, CH_3_), 2.50 (s, br., 2H, D_2_O-exchangeable, NH_2_), 2.81(s, 3H, CH_3_), 3.41 (s, 3H, CH_3_), 3.64 (s, 3H, CH_3_), 7.12 (s, br., 2H, D_2_O-exchangeable, NH_2_), 7.24-7.72 (m, 4H, Ar-H); MS *m/z* (%): 416 (M^+^, 4), 322 (13), 273 (12), 182 (61), 124 (100), 109 (99). Anal. Calcd. for C_17_H_20_N_8_OS_2_ (416.52): C, 49.02; H, 4.84; N, 26.90. Found C, 48.89; H, 4.76; N, 26.72%.

#### 3.1.3. Synthesis of 2-[(1-(2-Amino-4-methylthiazol-5-yl)ethylidene)hydrazono]-3,5-diphenyl-2,3-dihydro-1,3,4-thiadiazole (**11**)

A mixture of 1-[1-(2-amino-4-methylthiazol-5-yl)ethylidene]thiocarbohydrazide **3a** (0.244 g, 1 mmol) and *N*′-phenylbenzohydrazonoyl chloride **9** (0.230 g, 1 mmol) in dioxane (20 mL) containing triethylamine (0.1 g, 1 mmol) was refluxed for 6 h. The formed precipitate was isolated by filtration, washed with methanol, dried and recrystallized from DMF to give product **11**.

Yellow solid (76%); mp = 240–242 °C; IR (KBr): ν 3407, 3213 (NH_2_), 1634 (C=N) cm^−1^; ^1^H-NMR (DMSO-*d*_6_): δ 2.39 (s, 3H, CH_3_), 2.54 (s, 3H, CH_3_), 3.37 (s, br., 2H, D_2_O-exchangeable, NH_2_), 7.30–8.14 (m, 10H, Ar-H); MS *m/z* (%): 407 (M^+^ + 1, 67), 406 (M^+^, 52), 361 (59), 304 (64), 233 (91), 172 (64), 138 (67), 90 (79), 63 (100). Anal. Calcd. for C_20_H_18_N_6_S_2_ (406.53): C, 59.09; H, 4.46; N, 20.67. Found C, 59.01; H, 4.54; N, 20.45%.

#### 3.1.4. Alternative Synthesis of **11**

A mixture of 5-acetyl-2-amino-4-methylthiazole (**1**) (0.156 g, 1 mmol) and 2-hydrazono-3,5-diphenyl-2,3-dihydro-1,3,4-thiadiazole (**12**) (0.268 g, 1 mmol) in 10 mL of ethanol with catalytic amounts of glacial acetic acid were refluxed for 4h. The solid precipitated after cooling was filtered, washed with ethanol and recrystallized from acetic acid to give pure **11** which identical in all respects (m.p., mixed m.p. and IR spectra) with those obtained from reaction of **3a** with **9** but in 66% yield.

#### 3.1.5. Synthesis of 2-Amino-4-methyl-5-(1-(2-(4-methyl-5- (substitutedphenyldiazenyl)thiazol-2-yl)hydrazono)ethyl)thiazole (**14a**–**f**)

A mixture of 1-[1-(2-amino-4-methylthiazol-5-yl)ethylidene]thiosemicarbazide **3b** (0.229 g, 1 mmol) and appropriate hydrazonoyl halides **4a**–**f** (1 mmol) in dioxane (30 mL) containing triethylamine (0.1 g, 1 mmol) was refluxed for 6–8 h. (monitored by TLC). The formed precipitate after cooling was isolated by filtration, washed with methanol, dried and recrystallized from appropriate solvent to give products **14a**–**f**.

*2-Amino-4-methyl-5-(1-(2-(4-methyl-5-(phenyldiazenyl)thiazol-2-yl)hydrazono)ethyl)thiazole* (**14a**). Red solid (80%); mp 168–169 °C; IR (KBr): ν 3438, 3283 (NH_2_, NH), 1597 (C=N) cm^−1^; ^1^H-NMR (DMSO-*d*_6_): δ 2.43 (s, 3H, CH_3_), 2.56 (s, 3H, CH_3_), 3.56 (s, 3H, CH_3_), 6.95–7.35 (m, 5H, Ar-H), 7.57 (s, 2H, D_2_O-exchangeable, NH_2_), 10.51 (s, 1H, D_2_O-exchangeable, NH); ^13^C-NMR (DMSO-*d*_6_): δ 16.87 (CH_3_), 17.29 (CH_3_), 19.39 (CH_3_), 114.58, 119.46, 122.31, 129.69, 138.97, 144.14, 153.95 (Ar-C), 161.24, 168.59, 169.31, 176.75 (C=N); MS *m/z* (%): 372 (M^+^ + 1, 4), 371 (M^+^, 21), 218 (29), 153 (100), 78 (90), 42 (79). Anal. Calcd. for C_16_H_17_N_7_S_2_ (371.48): C, 51.73; H, 4.61; N, 26.39. Found C, 51.70; H, 4.46; N, 26.27%.

*2-Amino-4-methyl-5-(1-(2-(4-methyl-5-(methylphenyldiazenyl)thiazol-2-yl)hydrazono)ethyl)thiazole* (**14b**). Red solid (82%); mp 247–249 °C; IR (KBr): ν 3391, 3278 (NH_2_, NH), 1632 (C=N) cm^−1^; ^1^H-NMR (DMSO-*d*_6_): δ 2.25 (s, 3H, CH_3_), 2.43 (s, 3H, CH_3_), 2.53 (s, 3H, CH_3_), 3.56 (s, 3H, CH_3_), 7.13 (d, 2H, *J* = 6.9 Hz, Ar-H), 7.26 (d, 2H, *J* = 6.9 Hz, Ar-H), 7.55 (s, 2H, D_2_O-exchangeable, NH_2_), 10.44 (s, 1H, D_2_O-exchangeable, NH); ^13^C-NMR (DMSO-*d*_6_): δ 16.85 (CH_3_), 17.28 (CH_3_), 19.39 (CH_3_), 20.83 (CH_3_), 114.59, 119.48, 130.12, 131.28, 138.34, 141.83, 153.86 (Ar-C), 161.04, 168.69, 169.26, 176.58 (C=N); MS *m/z* (%): 386 (M^+^ + 1, 12), 385 (M^+^, 36), 232 (53), 153 (85), 113 (100). Anal. Calcd. for C_17_H_19_N_7_S_2_ (385.51): C, 52.96; H, 4.97; N, 25.43. Found C, 52.91; H, 4.86; N, 25.33%.

*2-Amino-4-methyl-5-(1-(2-(4-methyl-5-(chlorophenyldiazenyl)thiazol-2-yl)hydrazono)ethyl)thiazole* (**14c**). Red solid (75%); mp 178–180 °C; IR (KBr): ν 3996, 3283 (NH_2_, NH), 1632, 1606 (C=N) cm^−1^; ^1^H-NMR (DMSO-*d*_6_): δ 2.43 (s, 3H, CH_3_), 2.57 (s, 3H, CH_3_), 3.56 (s, 3H, CH_3_), 7.34 (s, 4H, Ar-H), 7.57 (s, 2H, D_2_O-exchangeable, NH_2_), 10.57 (s, 1H, D_2_O-exchangeable, NH); ^13^C-NMR (DMSO-*d*_6_): δ 16.87 (CH_3_), 17.29 (CH_3_), 19.43 (CH_3_), 116.04, 119.41, 125.79, 129.55, 139.81, 143.13, 154.26 (Ar-C), 161.53, 168.31, 169.38, 176.77 (C=N); MS *m/z* (%): 407 (M^+^ + 1, 5), 406 (M^+^, 18), 252 (18), 153 (99). 112 (95), 72 (100). Anal. Calcd. for C_16_H_16_ClN_7_S_2_ (405.93): C, 47.34; H, 3.97; N, 24.15. Found C, 47.39; H, 3.90; N, 24.06%.

*2-Amino-4-methyl-5-(1-(2-(4-methyl-5-(bromophenyldiazenyl)thiazol-2-yl)hydrazono)ethyl)thiazole* (**14d**). Red solid (75%); mp 176–178 °C; IR (KBr): ν 3368, 3191 (NH_2_, NH), 1632 (C=N) cm^−1^; ^1^H-NMR (DMSO-*d*_6_): δ 2.42 (s, 3H, CH_3_), 2.53 (s, 3H, CH_3_), 3.59 (s, 3H, CH_3_), 7.28 (d, 2H, *J* = 4.5 Hz, Ar-H), 7.48(d, 2H, *J* = 4.5 Hz, Ar-H), 7.58 (s, 2H, D_2_O-exchangeable, NH_2_), 10.58 (s, 1H, D_2_O-exchangeable, NH); ^13^C-NMR (DMSO-*d*_6_): δ 16.87 (CH_3_), 17.30 (CH_3_), 19.43 (CH_3_), 113.65, 116.48, 119.42, 132.55, 139.91, 143.53, 154.25 (Ar-C), 161.53, 168.29, 169.38, 176.75 (C=N); MS *m/z* (%): 452 (M^+^ + 1, 2), 451 (M^+^, 15), 296 (14), 153 (100), 113 (86), 72 (98). Anal. Calcd. for C_16_H_16_BrN_7_S_2_ (450.38): C, 42.67; H, 3.58; N, 21.77. Found C, 42.55; H, 3.67; N, 21.65%.

*2-Amino-4-methyl-5-(1-(2-(4-methyl-5-(methoxylphenyldiazenyl)thiazol-2-yl)hydrazono)ethyl)thiazole* (**14e**). Red solid (78%); mp 187–189 °C; IR (KBr): ν 3414, 3202 (NH_2_, NH), 1624 (C=N) cm^−1^; ^1^H-NMR (DMSO-*d*_6_): δ 2.43 (s, 3H, CH_3_), 2.53 (s, 3H, CH_3_), 3.56 (s, 3H, CH_3_), 3.72 (s, 3H, OCH_3_), 6.91–7.30 (m, 4H, Ar-H), 7.87 (s, 2H, D_2_O-exchangeable, NH_2_), 10.48 (s, 1H, D_2_O-exchangeable, NH); ^13^C-NMR (DMSO-*d*_6_): δ 8.93 (CH_3_), 16.85 (CH_3_), 17.19 (CH_3_), 55.73 (OCH_3_), 115.07, 115.87, 119.38, 137.60, 137.74, 155.30, 160.59 (Ar-C), 169.08, 169.21, 169.26, 176.60 (C=N); MS *m/z* (%): 402 (M^+^ + 1, 2), 401 (M^+^, 13), 248 (17), 153 (38), 113 (42), 44 (100). Anal. Calcd. for C_17_H_19_N_7_OS_2_ (401.51): C, 50.85; H, 4.77; N, 24.42. Found C, 50.82; H, 4.65; N, 24.23%.

*2-Amino-4-methyl-5-(1-(2-(4-methyl-5-(nitrophenyldiazenyl)thiazol-2-yl)hydrazono)ethyl)thiazole* (**14f**). Red solid (78%); mp 183–185°C; IR (KBr): *v* 3375, 3194 (NH_2_, NH), 1643 (C=N) cm^−1^; ^1^H-NMR (DMSO-*d*_6_): δ 2.42 (s, 3H, CH_3_), 2.54 (s, 3H, CH_3_), 3.57 (s, 3H, CH_3_), 6.91–7.38 (m, 4H, Ar-H), 7.59 (s, 2H, D_2_O-exchangeable, NH_2_), 10.53 (s, 1H, D_2_O-exchangeable, NH); ^13^C-NMR (DMSO-*d*_6_): δ 16.45 (CH_3_), 17.29 (CH_3_), 19.76 (CH_3_), 114.54, 119.53, 122.31, 129.60, 138.91, 144.23, 153.92 (Ar-C), 161.34, 168.55, 169.36, 176.75 (C=N); MS *m/z* (%): 417 (M^+^ + 1, 3), 416 (M^+^, 3), 153 (33), 113 (24), 43 (100). Anal. Calcd. for C_16_H_16_N_8_O_2_S_2_ (416.48): C, 46.14; H, 3.87; N, 26.90. Found C, 46.11; H, 3.76; N, 26.64%.

#### 3.1.6. Synthesis of Methyl 2-(2-((1-(2-amino-4-methylthiazol-5-yl)ethylidene)hydrazono)-4-oxothiazolidin-5-ylidene)acetate (**17**)

To a solution of 1-[1-(2-amino-4-methylthiazol-5-yl)ethylidene]thiosemicarbazide **3b** (0.229 g, 1 mmol) in dry methanol (20 mL) was added dimethylacetylenedicarboxylate **15** (0.142 g, 1 mmol). The solution was refluxed for 2 h. The precipitated product after cooling was filtered, washed with methanol, and recrystallized from ethanol to give product **17**.

Canary yellow solid (75%); mp 352–354 °C; IR (KBr): *v* 3306, 3167 (NH_2_, NH), 1708, 1689 (2C=O), 1609 (C=N) cm^−1^; ^1^H-NMR (DMSO-*d*_6_): δ 2.39 (s, 3H, CH_3_), 2.50 (s, 3H, CH_3_), 3.76 (s, 3H, OCH_3_), 6.60 (s, 1H, =C*H*COOCH_3_), 9.43 (s, 2H, D_2_O-exchangeable, NH_2_), 12.88 (s, 1H, D_2_O-exchangeable, NH); ^13^C-NMR (DMSO-*d*_6_): δ 15.03 (CH_3_), 16.01 (CH_3_), 52.92 (OCH_3_), 114.64, 118.82, 139.60 143.31 (Ar-C), 157.67, 160.07, 165.97 (C=N), 168.37, 168.37 (C=O); MS *m/z* (%): 339 (M^+^, 68), 153 (53), 113 (38), 85 (100), 43 (57). Anal. Calcd. for C_12_H_13_N_5_O_3_S_2_ (339.39): C, 42.47; H, 3.86; N, 20.63. Found C, 42.40; H, 3.89; N, 20.43%.

### 3.2. Biological Assay

#### 3.2.1. WST-1 Assay

The human hepatocellular carcinoma cell line were cultured and tested at Nanotechnology & Advanced materials central lab, Giza, Egypt. The culture was maintained in DMEM with 10% FBS at 37 °C humidified with 5% CO_2_. Various concentrations of the compound being test, as well as doxorubicin as a reference drug (0.0, 0.04, 0.1, 0.2, 0.3, 0.4, and 0.6 μg/mL), were added to the cell monolayer in triplicate wells; then, the individual doses and their cytotoxicity were tested using a standard WST-1 cell proliferation assay as a fast and sensitive quantification of cell proliferation and viability in a 96-well microtiter plate for 24 h, measuring the absorbance of the dye solution at 450 nm [35].

#### 3.2.2. Confocal Laser Scanning Microscopy (CLSM)

The Mode of Potential cytotoxicity action was evaluated using confocal laser scanning microscopic (Carrl Zeiss CLSM 710, diverse net ventures, Boston, MA, USA) imaging of Hep G2 treated cell lines at 0.6 μg/mL concentration of the tested compounds. Cells were plated in 96-multiwill plates (10^4^ cells/well) for 24 h before treatment with the tested compound to allow attachment of cells to the wall of the plate. A selected concentration of the compounds being tested (0.6 μM) was added to the cell monolayer in triplicate wells for each individual dose; monolayer cells were incubated with the compounds for 24 h at 37 °C and in atmosphere of 5% CO_2_. After 24 h, cells were stained by Rhodamine 132 and Acridine orange stains (Sigma-Aldrich, Boston, MA, USA); after further waiting for five minutes, microscopic examination was done using excitation laser lines at 588 nm and 633 nm by two channel detection.

### 3.3. Molecular Docking Using Leadit 2.1.5

All compounds were built and saved as Mol2. The crystal structure of human LDH-5B in complex with nicotinamide adenine dinucleotide was downloaded from protein data bank (pdb code = 1T2F). The protein was loaded into Leadit 2.1.5 and the receptor components were chosen by selection of chain A as a main chain. Binding site was defined by choosing NAD^+^ as a reference ligand to which all coordinates were computed. Amino acids within radius 6.5 A° were selected in the binding site. All chemical ambiguities of residues were left as default. Ligand binding was driven by enthalpy (classic Triangle matching). For scoring, all default settings were restored. Intra-ligand clashes were computed using clash factor = 0.6. Maximum number of solutions per iteration = 200. Maximum number of solutions per fragmentation = 200. The base placement method was used as a docking strategy.

## 4. Conclusions

New thiazole derivatives have been synthesized using ethylidenethiosemicarbazide and ethylidenethiocarbohydrazide as starting materials under thermal conditions. Compounds **14e**, **14c** and **14a** may have significant and promising anticancer efficiency for hepatocellular carcinoma with low IC_50_, 0.5 ± 0.02, 0.52 ± 0.03, and 0.84 ± 0.04 μM, respectively. The cytotoxic effect was due to its inhibitory effect to the inner mitochondrial membrane Lactate dehydrogenase enzyme, which, in turn, decreases cellular activity, including the rate of cell division. The molecular docking study confirmed high binding affinities of –24.85, –24.23, and –24.10 kcal/mol for **14e**, **14c** and **14a**, respectively. A direct correlation between the computed affinity and the IC_50_ was observed.

## Supplementary Materials

Supplementary materials can be accessed at: http://www.mdpi.com/1420-3049/21/01/0003/s1.
