# Supplementary material for: Synthesis, Characterization and Molecular Docking of Novel Bioactive Thiazolyl-Thiazole Derivatives as Promising Cytotoxic Antitumor Drug
Source: Molecules. 2015 Dec 22;21(1):3. doi: 10.3390/molecules21010003 (PMC6272888; doi:10.3390/molecules21010003)
Supplement: Supplementary file 1 [file molecules-21-00003-s001.pdf]

Supplementary Materials: Synthesis, Characterization and Molecular Docking of Novel Bioactive Thiazolyl-Thiazole Derivatives as promising Cytotoxic Antitumor Drug

Sobhi M. Gomha, Taher A. Salah, Huwaida M. E. Hassaneen and Mohammed A. Khedr

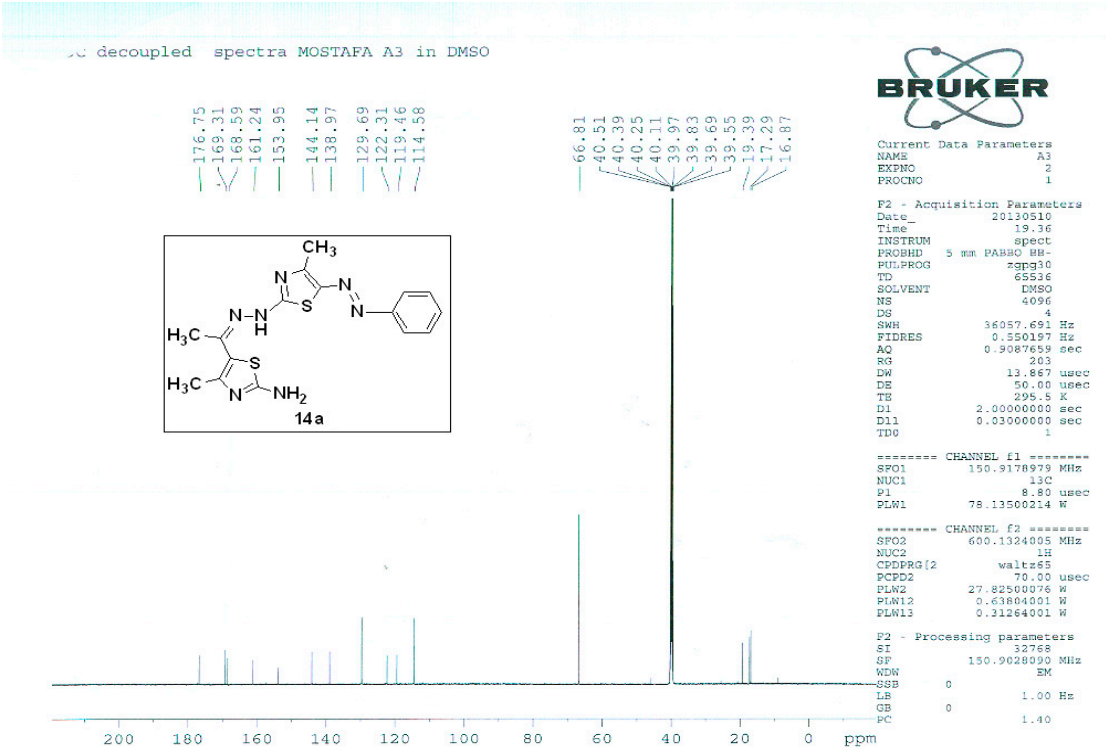

Figure S1. ^{13}C-NMR of compound **14a**.

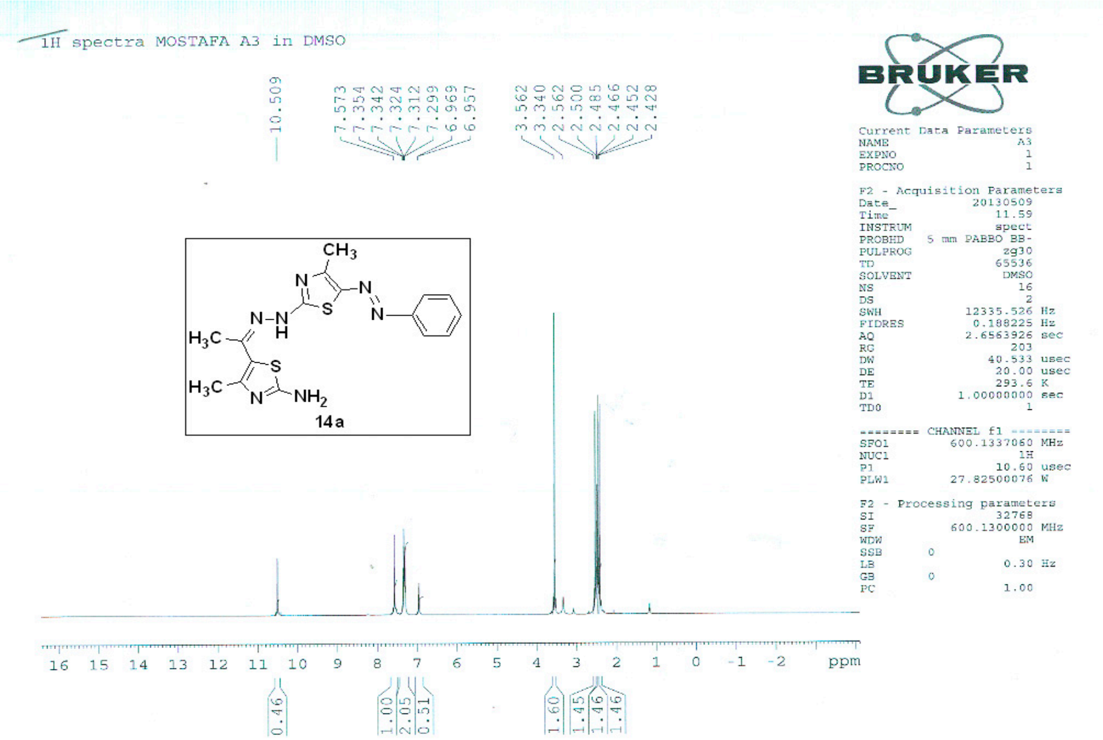

Figure S2. ^{1}H-NMR of compound **14a**.

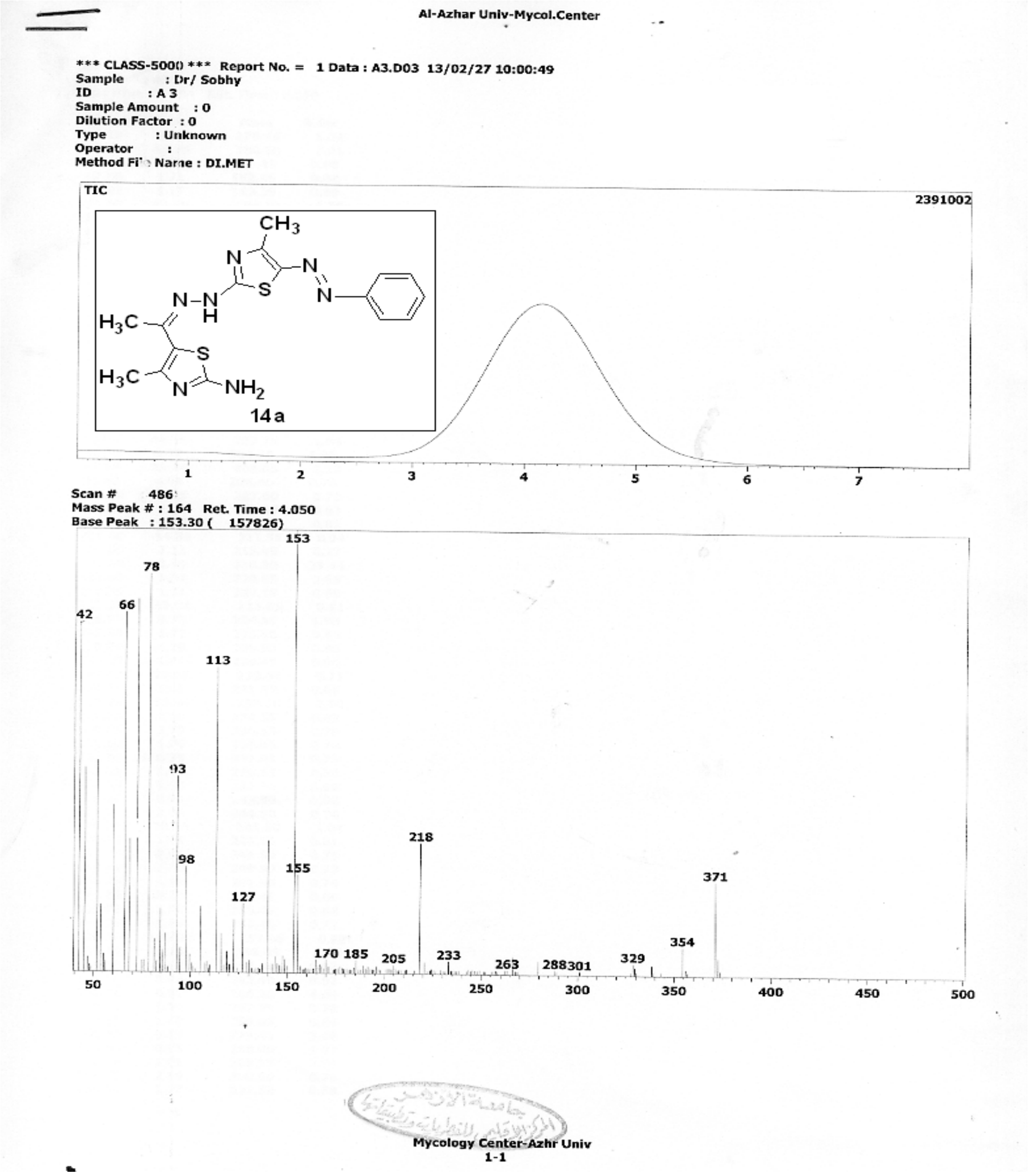

Figure S3. Mass of compound **14a**.

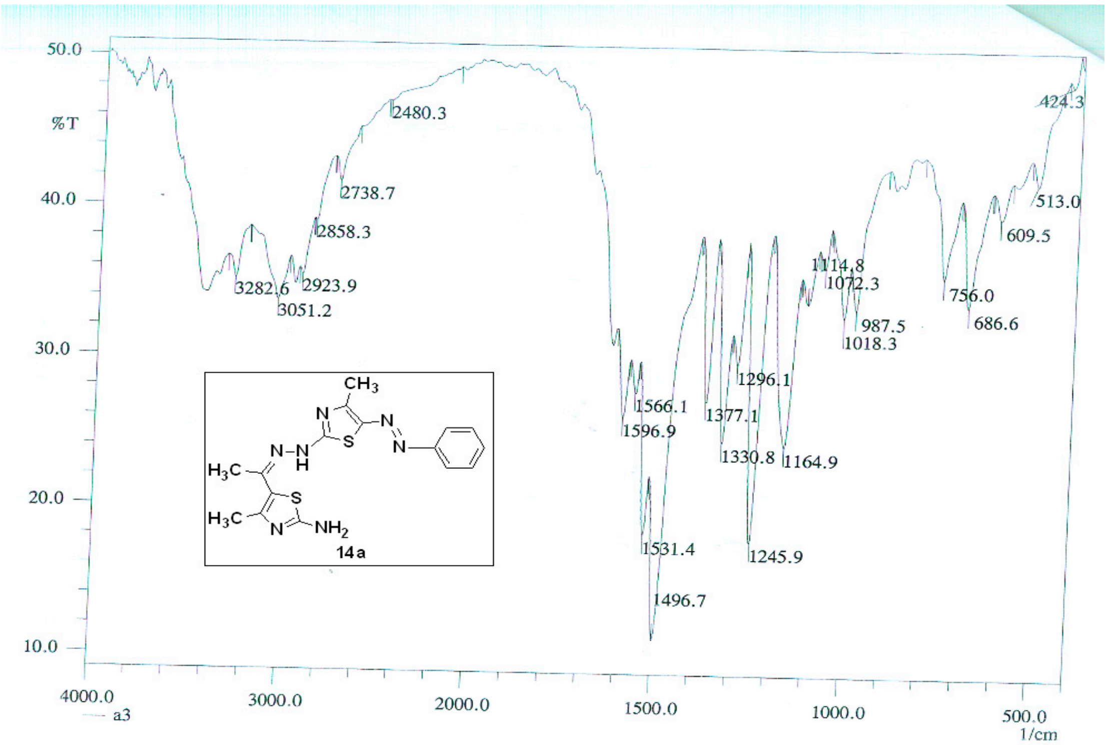

Figure S4. IR of compound **14a**.

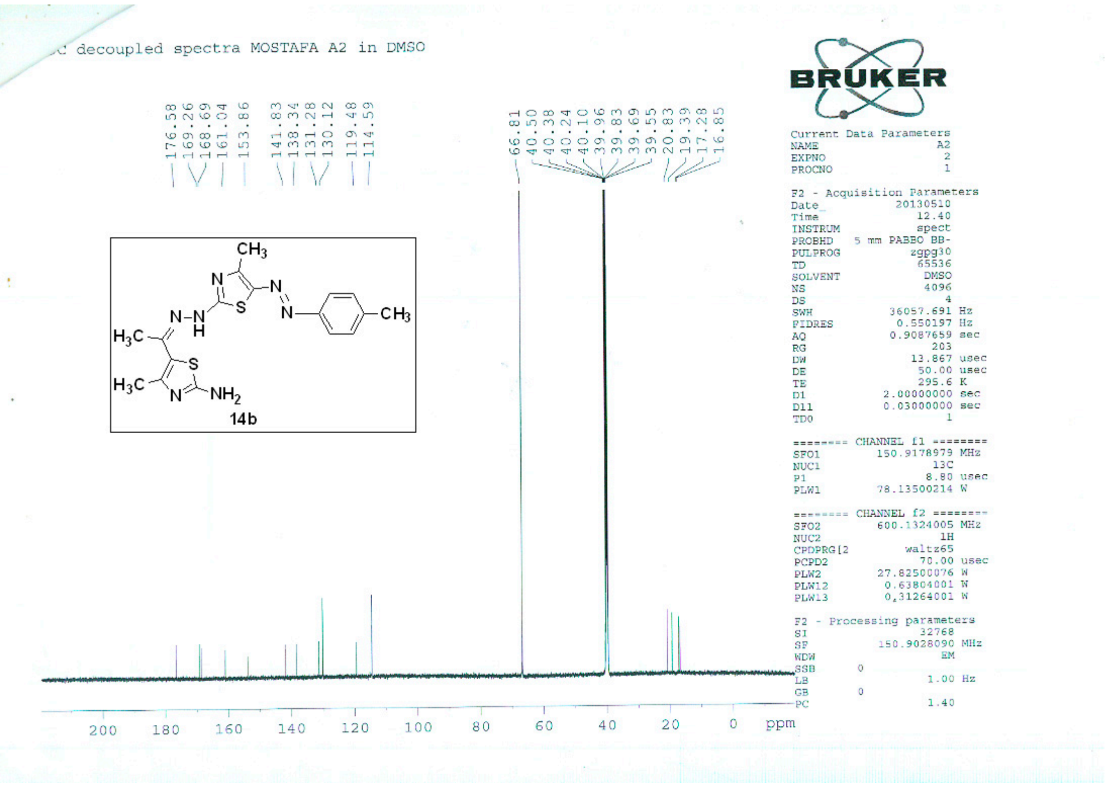

Figure S5. ^{13}C-NMR of compound **14b**.

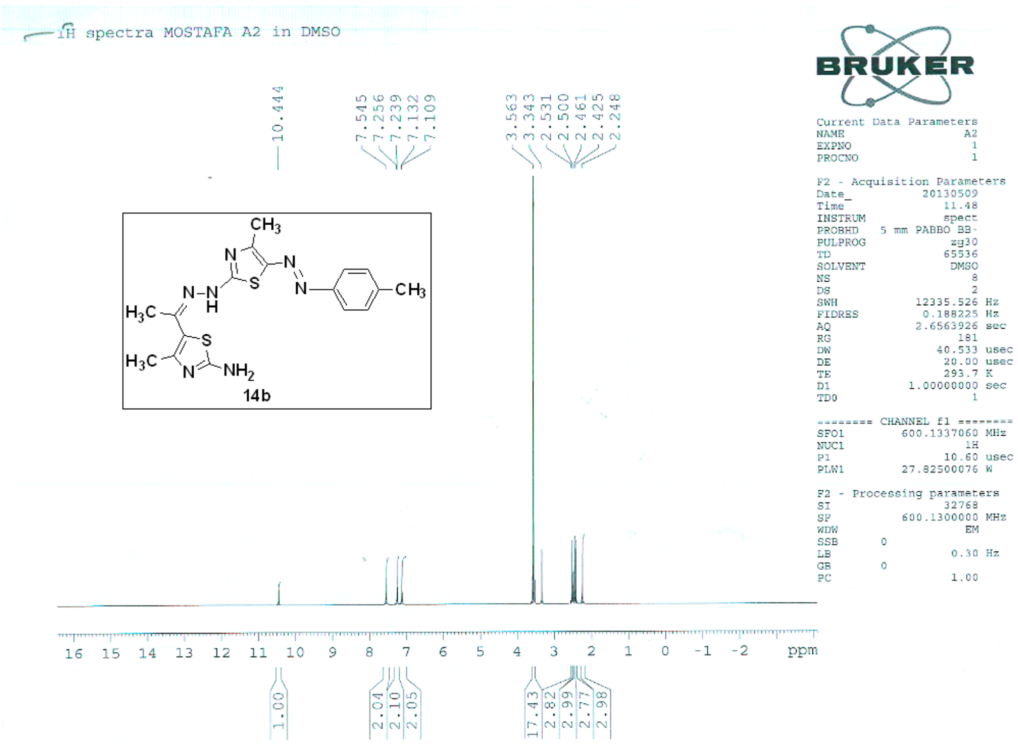

Figure S6. ^{1}H-NMR of compound **14b**.

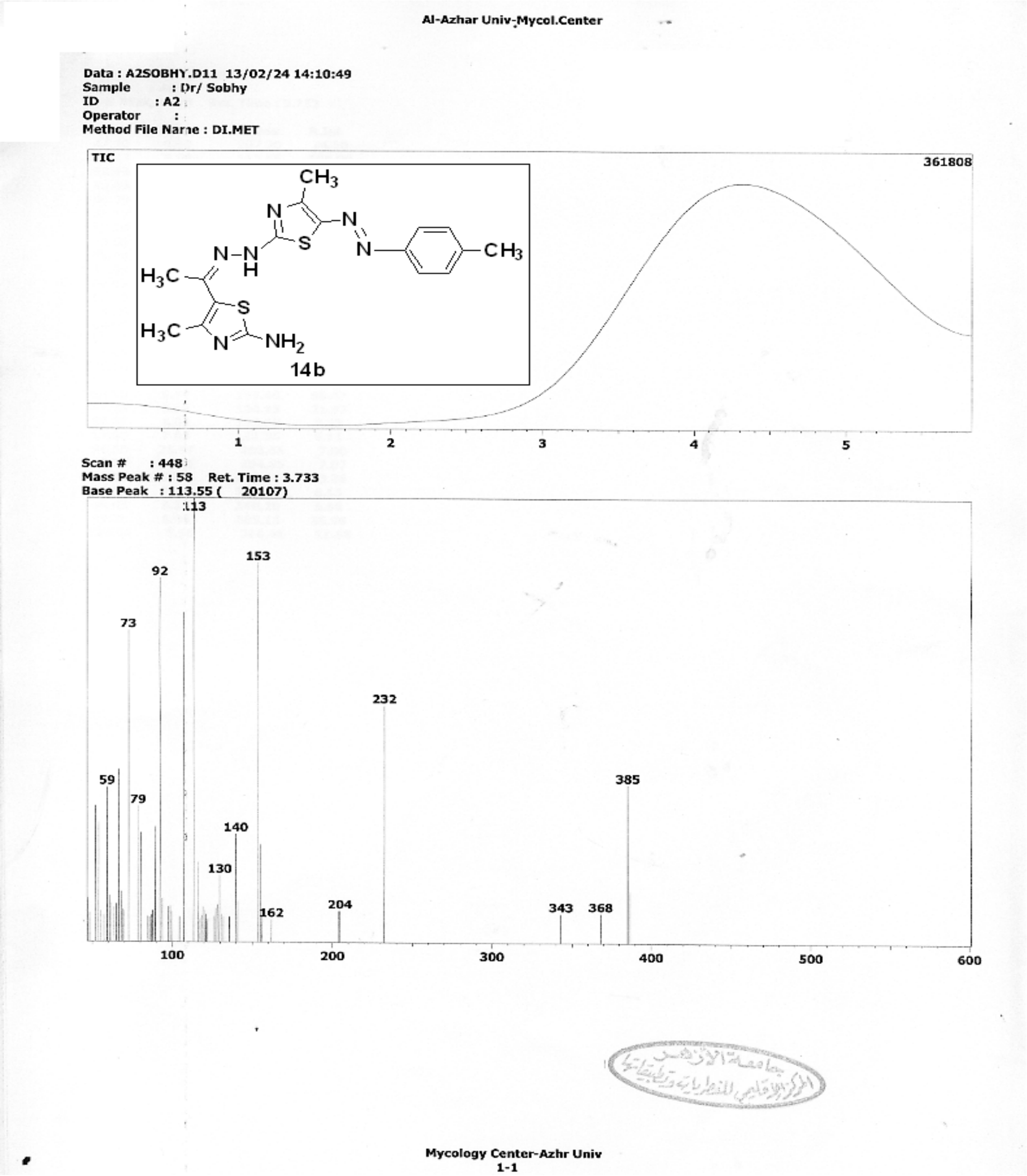

Figure S7. Mass of compound **14b**.

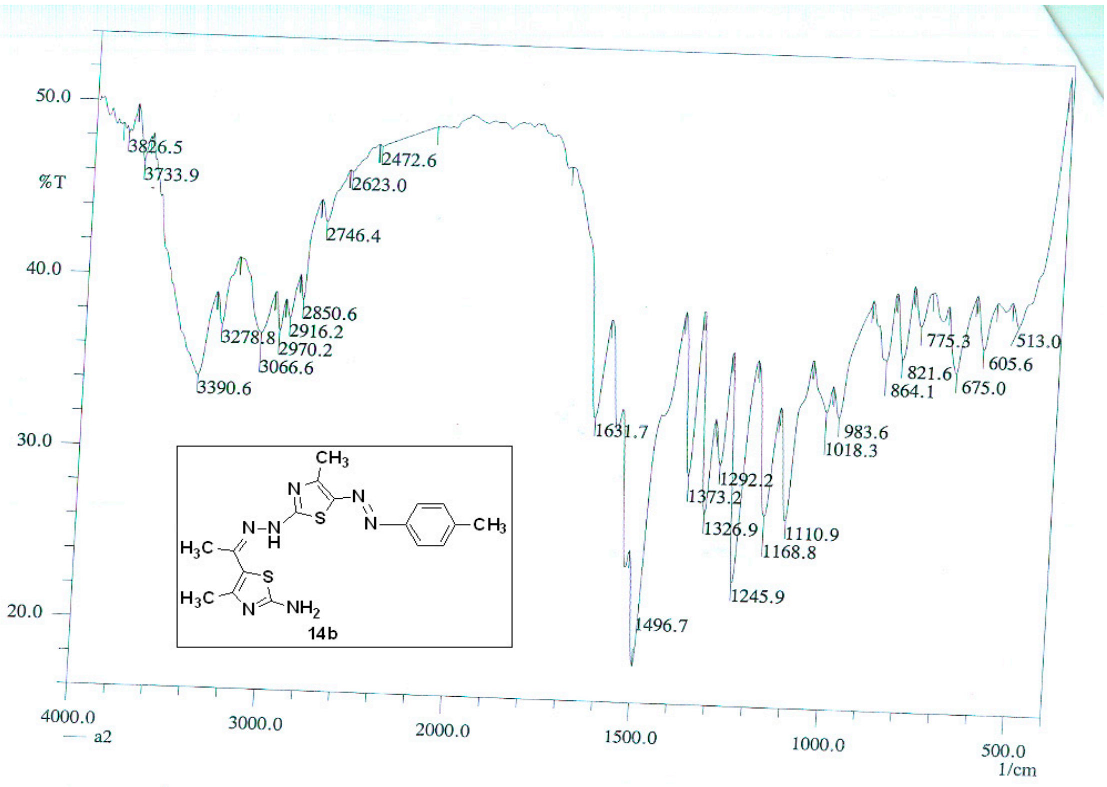

Figure S8. IR of compound **14b**.

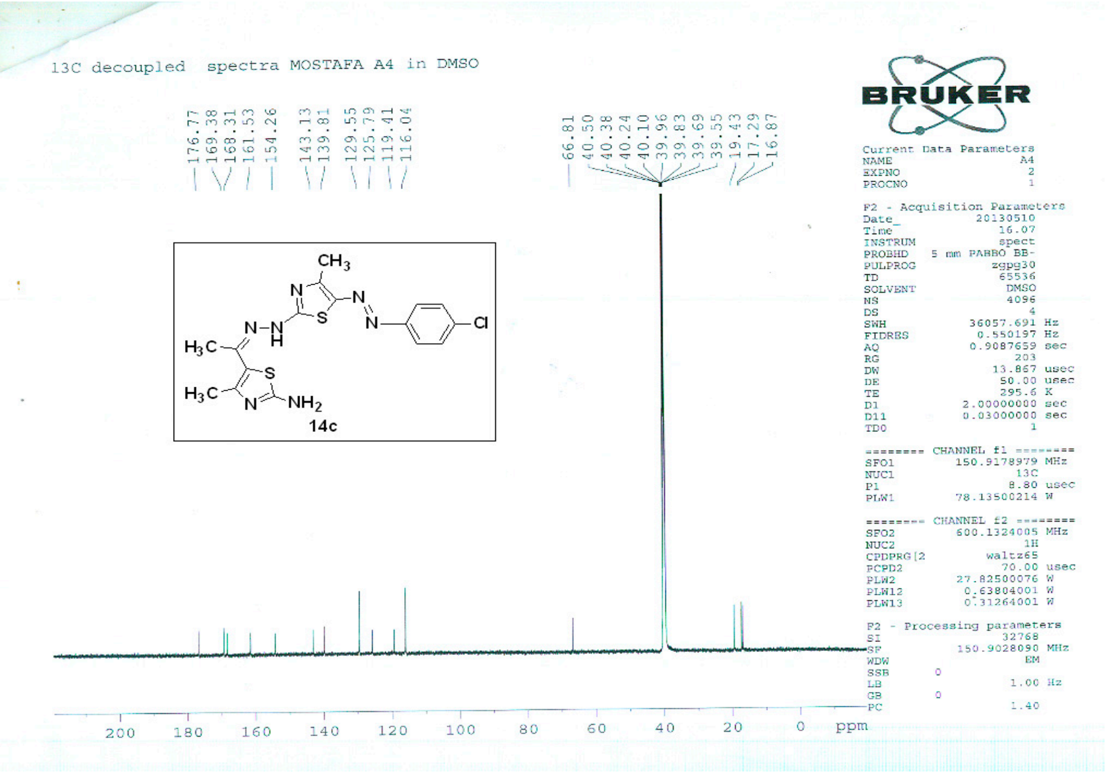

Figure S9. ^{13}C-NMR of compound **14c**.

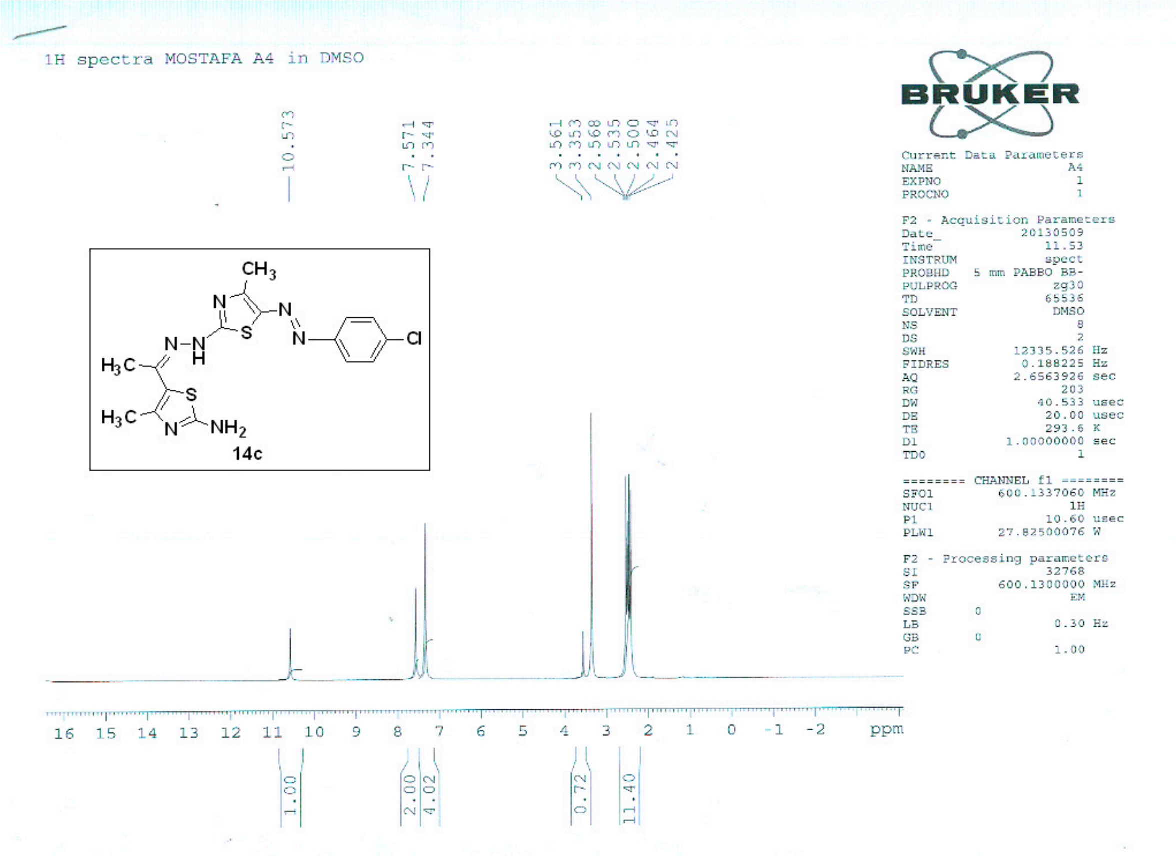

Figure S10. ^{1}H-NMR of compound **14c**.

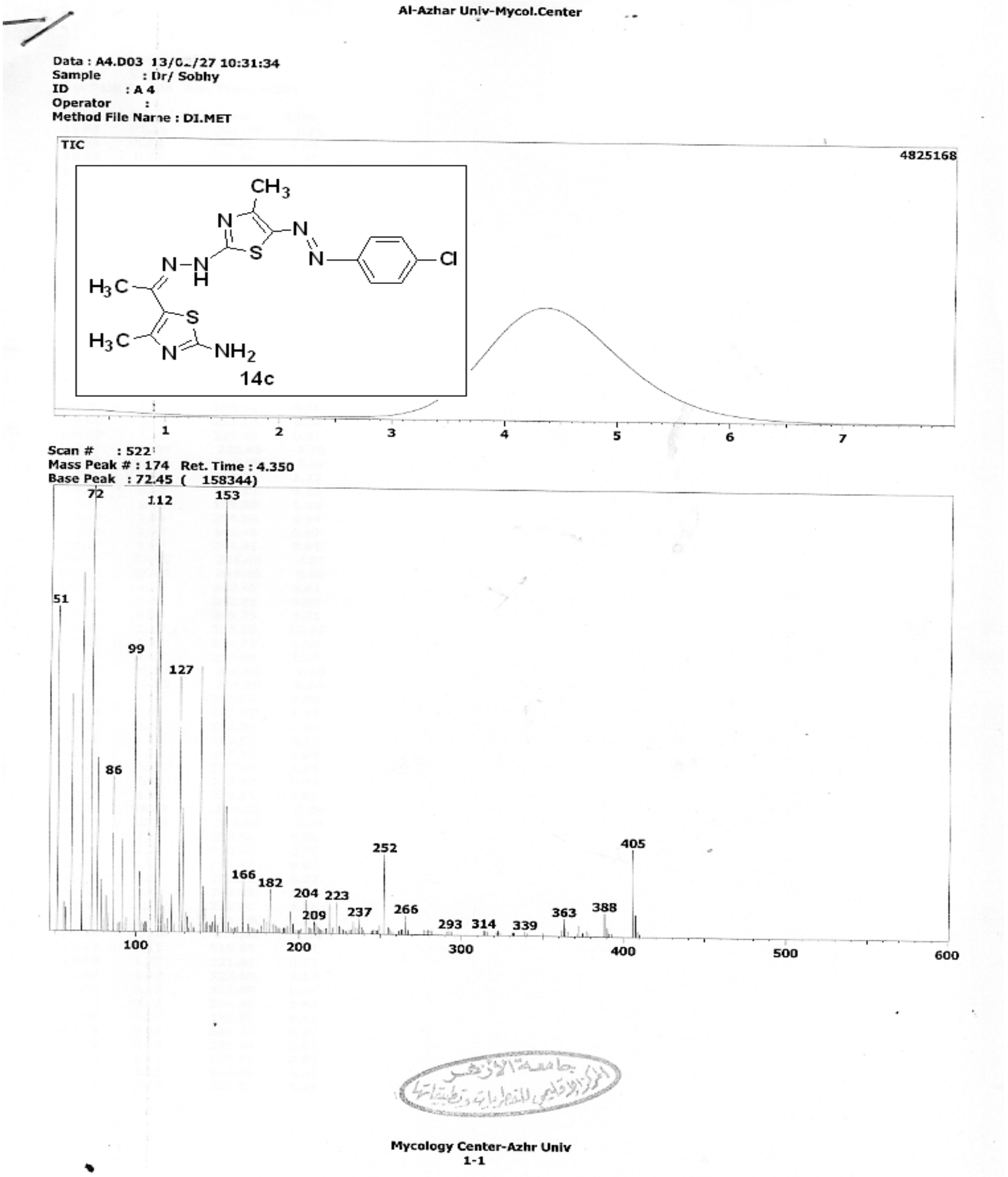

Figure S11. Mass of compound **14c**.

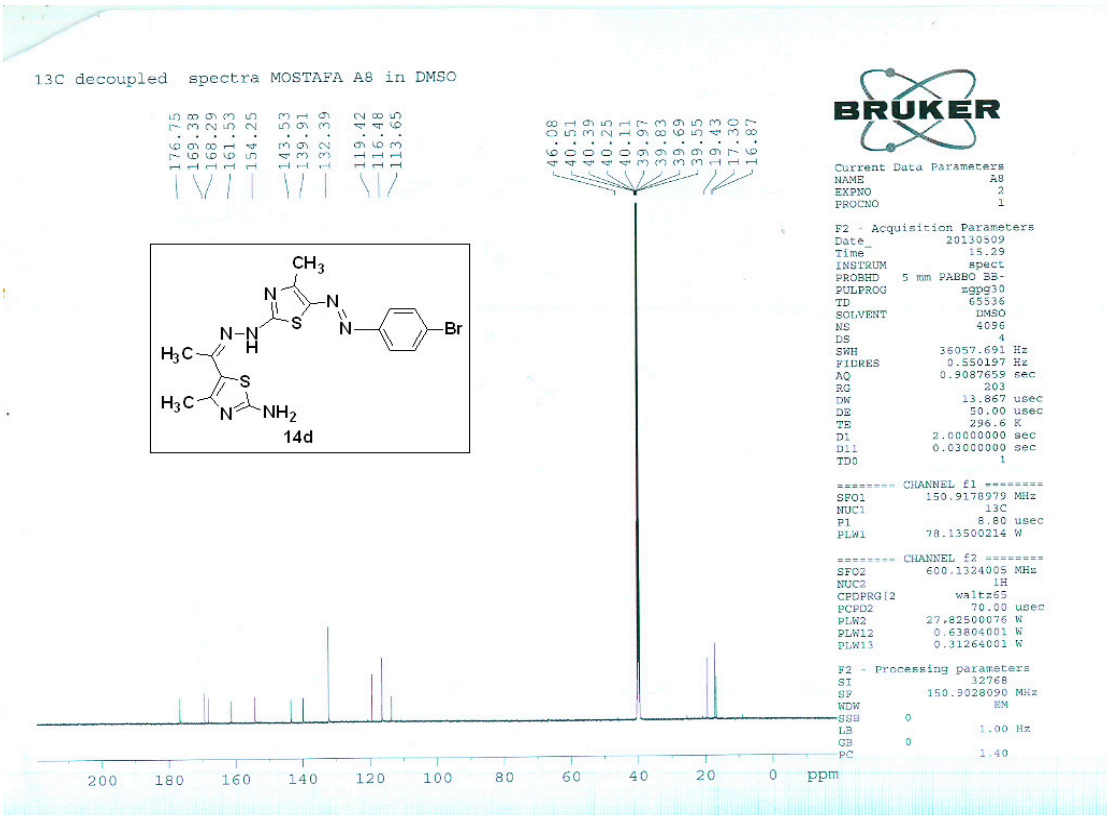

Figure S12. ^{13}C-NMR of compound **14d**.

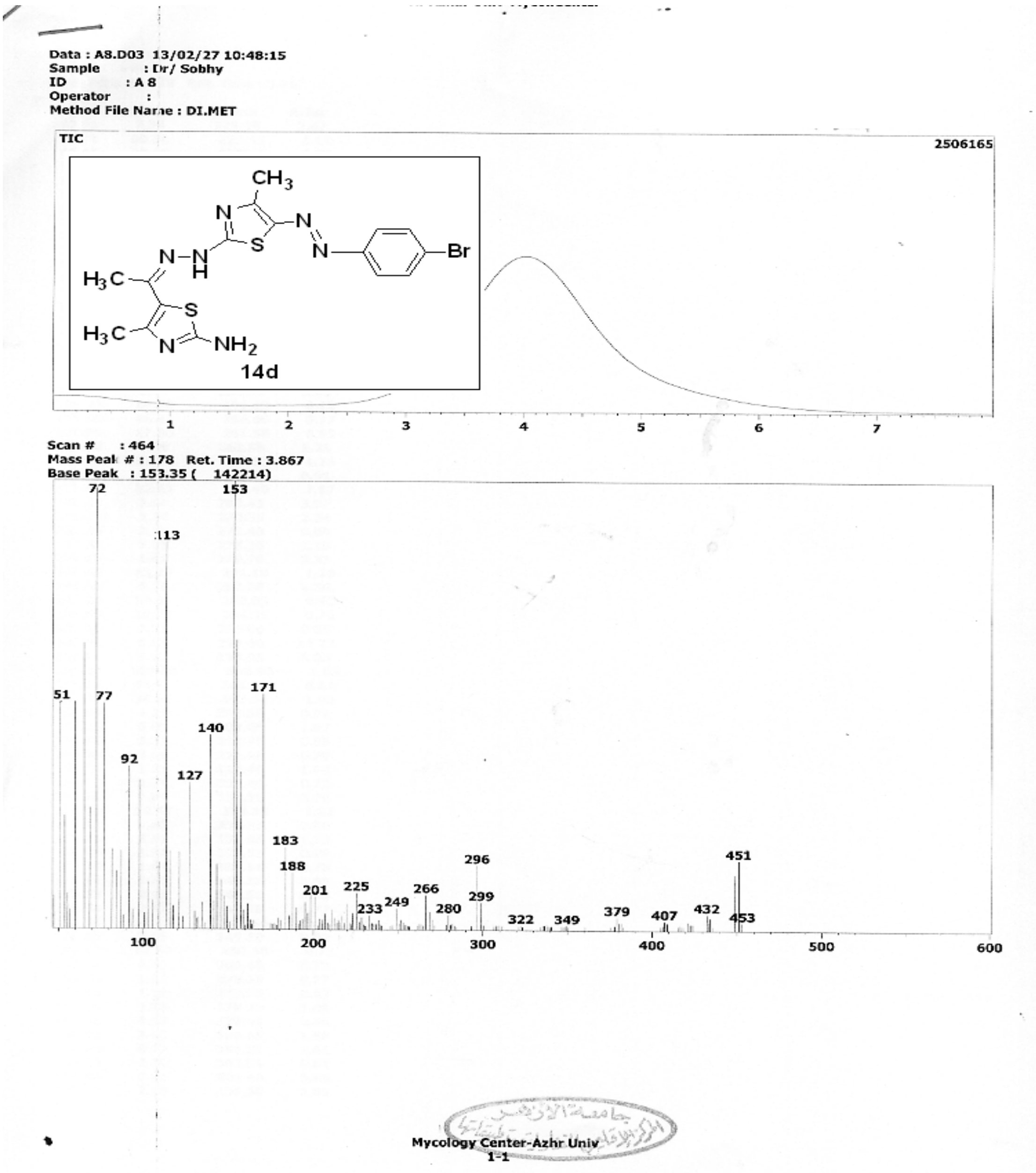

Figure S13. Mass of compound **14d**.

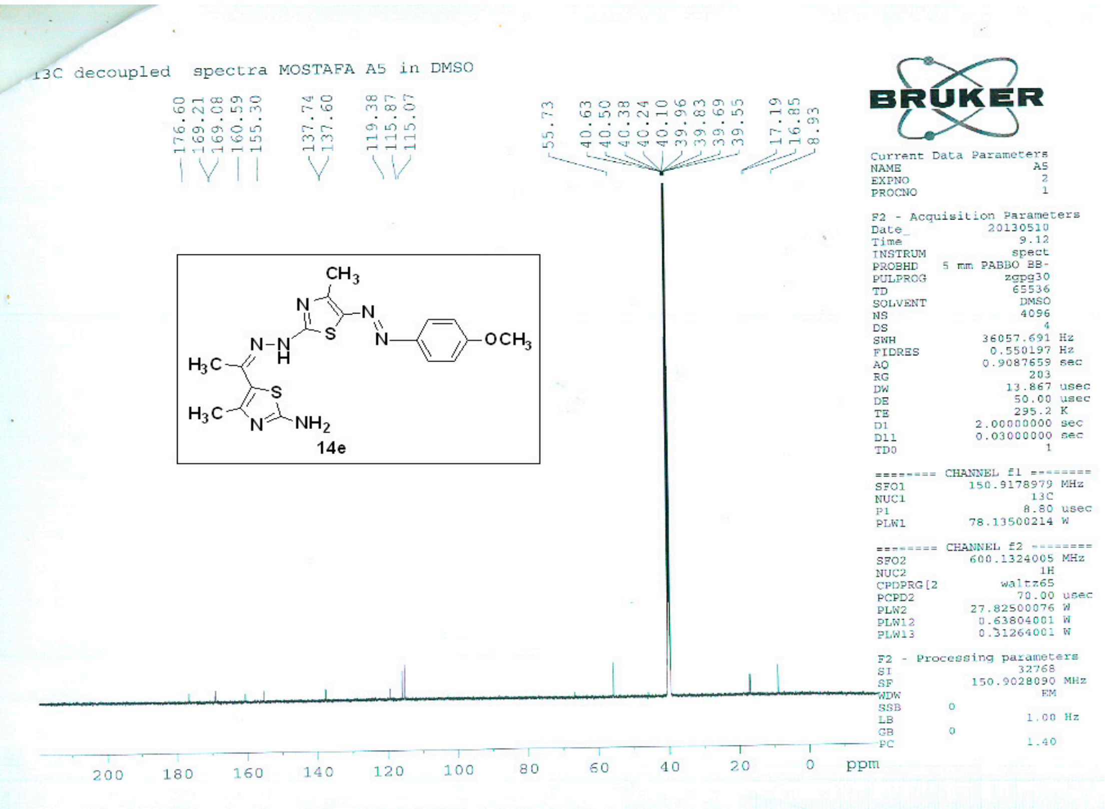

Figure S14. ^{13}C-NMR of compound **14e**.

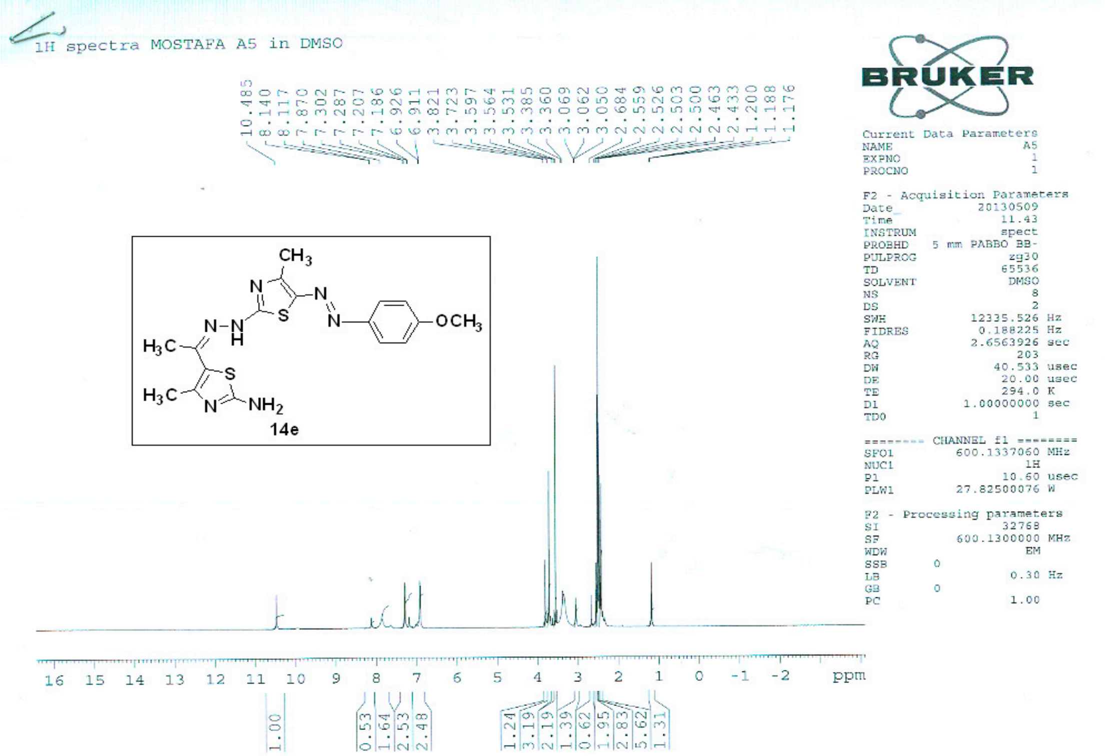

Figure S15. ^{1}H-NMR of compound **14e**.

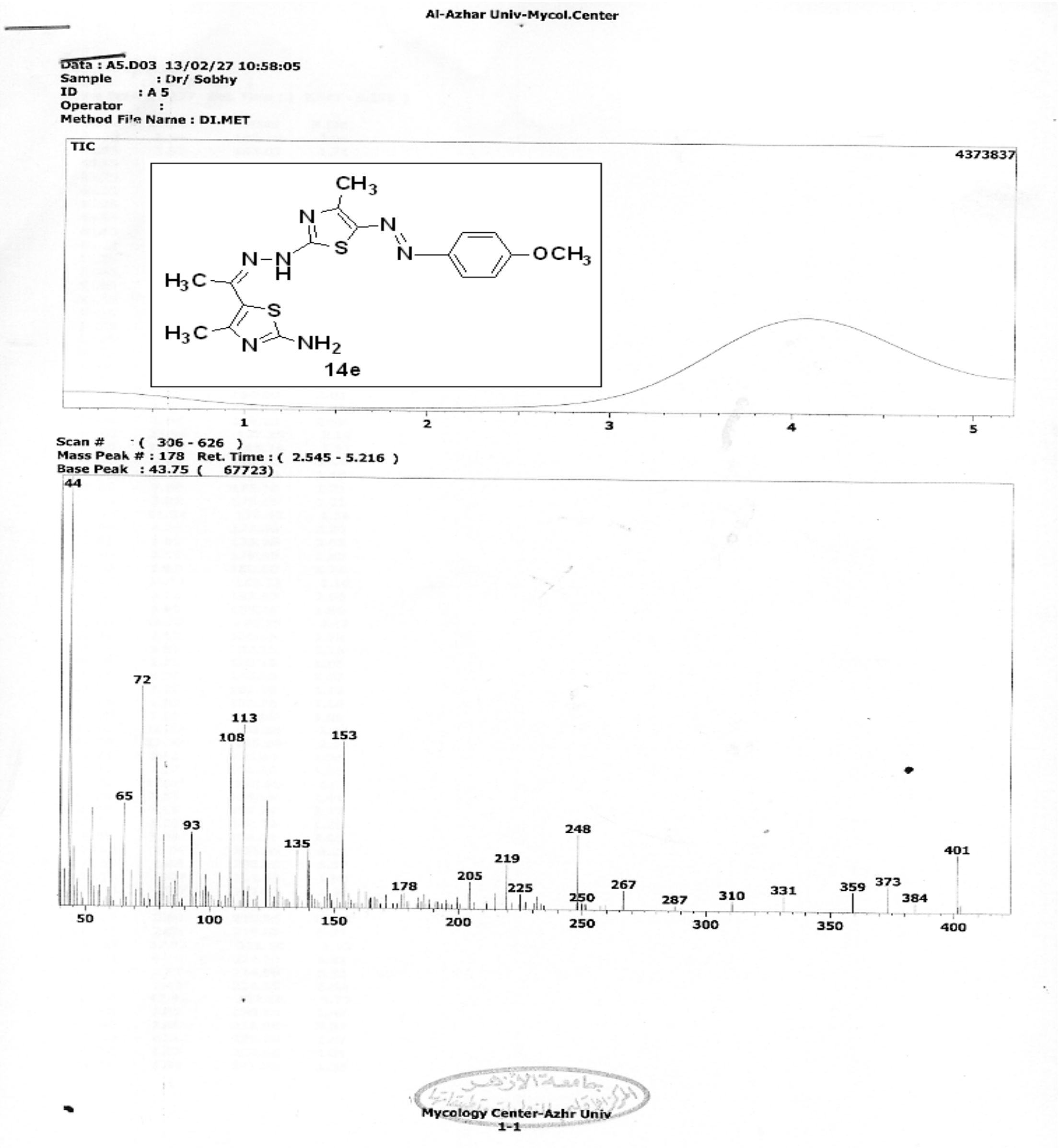

Figure S16. Mass of compound **14e**.

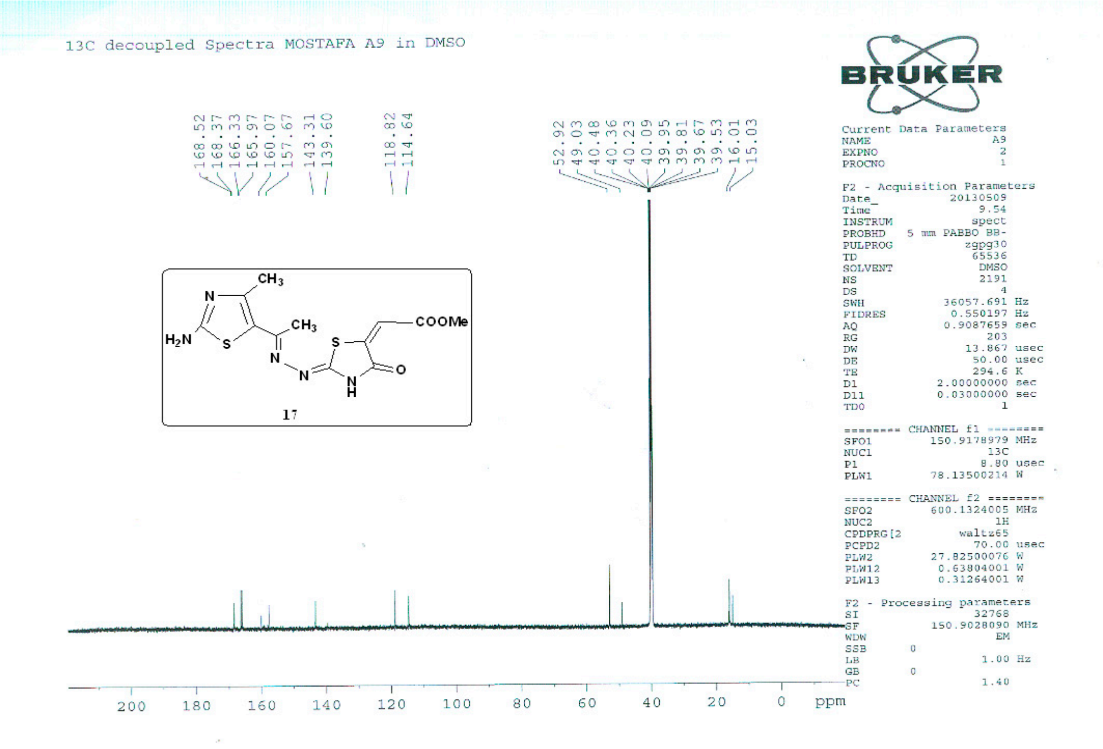

Figure S17. ^{13}C-NMR of compound **17**.

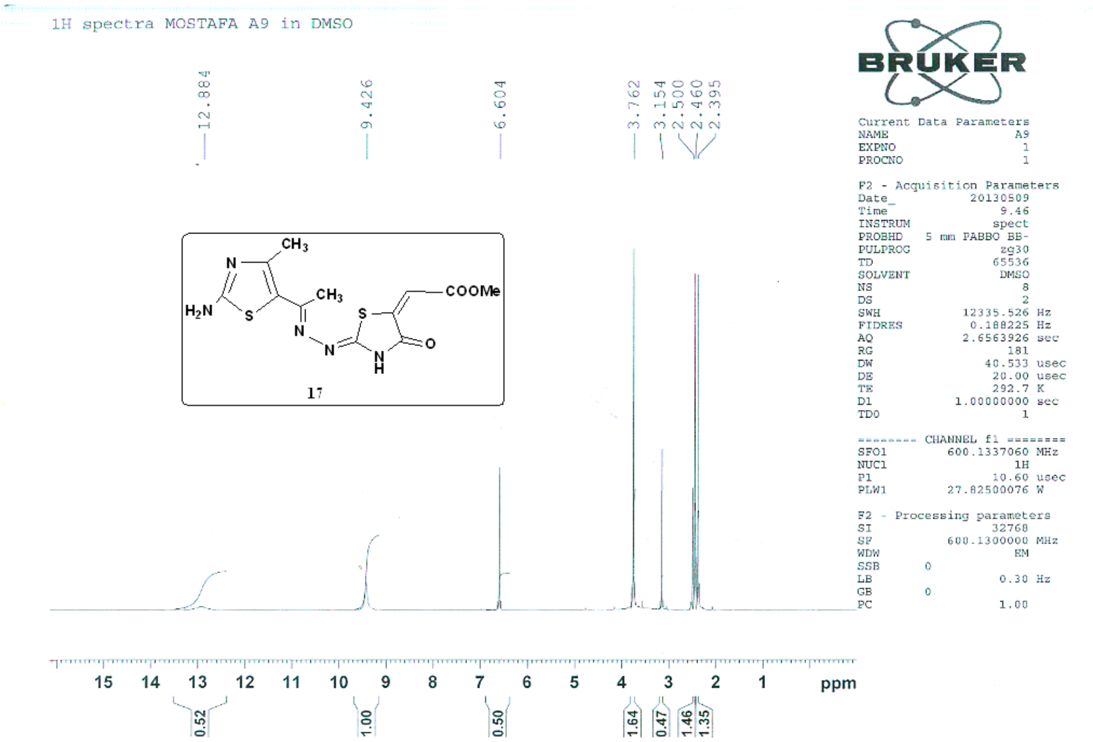

Figure S18. ^{1}H-NMR of compound **14e**.

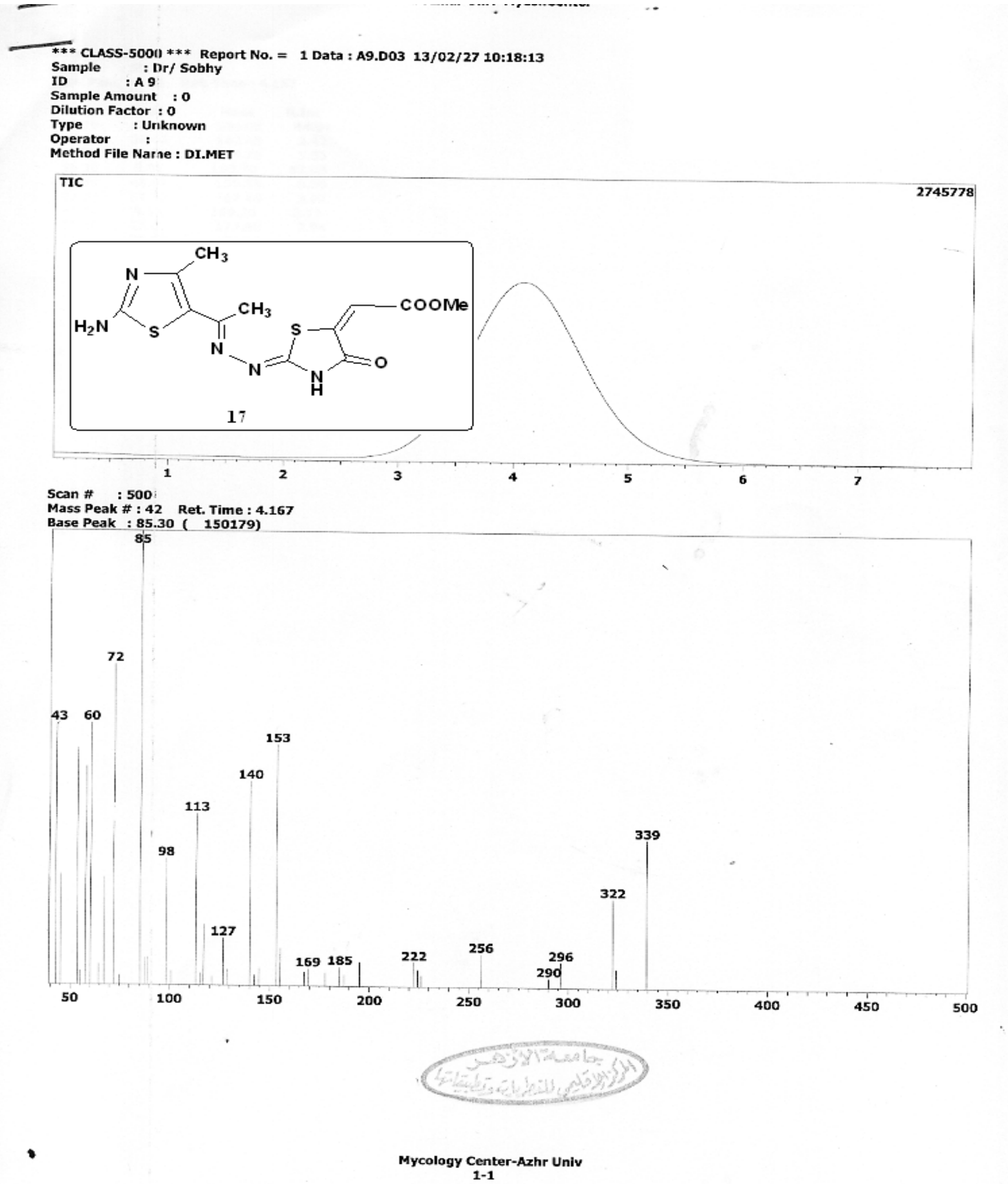

Figure S19. Mass of compound **17**.

15-Sep-13 13:52:36

Cairo University
Micro Analytical Center

DI Analysis
Shimadzu Qp-2010 Plus

Sample Information

Analyzed by	: A.GABR
Analyzed	: 15/09/2013 01:44:16 م
Sample Name	: A22
Sample ID	:
Customer Name	: صبحي محمد - علوم القاهره
Data File	: C:\GCMSsolution\Data\Project1\A22.QGD
Org Data File	: C:\GCMSsolution\Data\Project1\A22.QGD
Method File	: C:\GCMSsolution\Data\Project1\A.GABR.qgm
Org Method File	: C:\GCMSsolution\Data\Project1\A.GABR.qgm
Report File	:
Tuning File	: C:\GCMSsolution\System\Tune1_default1.qgt
$EndIf$Modified by	: A.GABR
Modified	: 15/09/2013 01:49:45 م

Method

==== Analytical Line 1 ====

IonSourceTemp	:250.00 °C
[MS Table]	
--Group 1 - Event 1--	
Start Time	:0.00min
End Time	:10.00min
ACQ Mode	:Scan
Event Time	:0.50sec
Scan Speed	: 526
Start m/z	:50.00
End m/z	:300.00
Electron Voltage	: 70 eV
Ionization Mode	: EI

C:\GCMSsolution\Data\Project1\A22.QGD

Chromatogram A22 C:\GCMSsolution\Data\Project1\A22.QGD

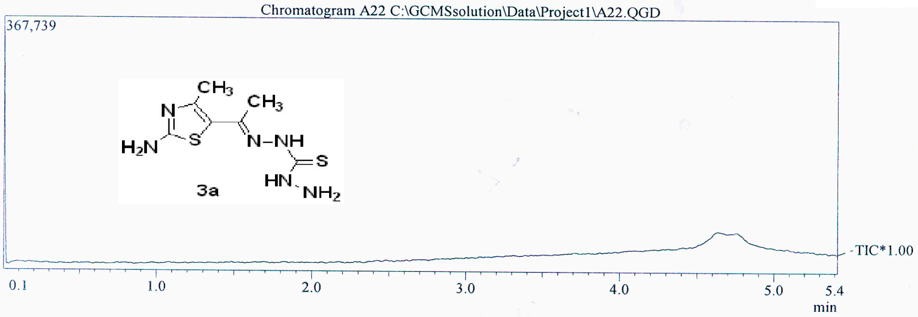

Spectrum

Line#:1 R.Time:3.5(Scan#:426)
MassPeaks:66
RawMode:Single 3.5(426) BasePeak:79(272)
BG Mode:Averaged 3.5-3.5(422-422) Group 1 - Event 1

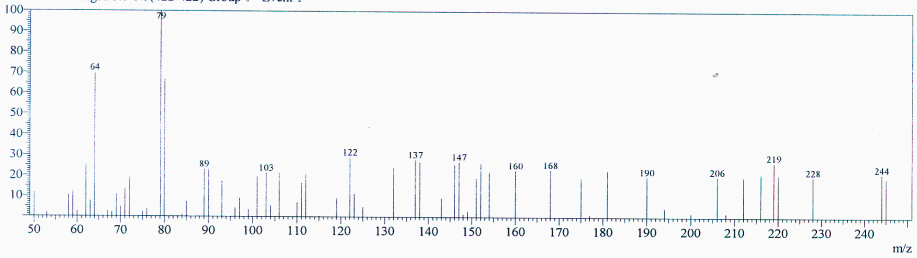

Mass Table
Line#:1 R.Time:3.5(Scan#:426)
MassPeaks:66
RawMode:Single 3.5(426) BasePeak:79(272)
BG Mode:Averaged 3.5-3.5(422-422) Group 1 - Event 1

#	m/z	Abs. In	Rel. Int.	#	m/z	Abs. In	Rel. Int.	#	m/z	Abs. In	Rel. Int.
1	50.00	32	11.76	4	59.00	33	12.13	7	63.00	21	7.72
2	53.00	6	2.21	5	60.00	7	2.57	8	63.95	190	69.85
3	58.00	29	10.66	6	62.00	68	25.00	9	67.00	7	2.57

Figure S20. Mass of compound **3a**.

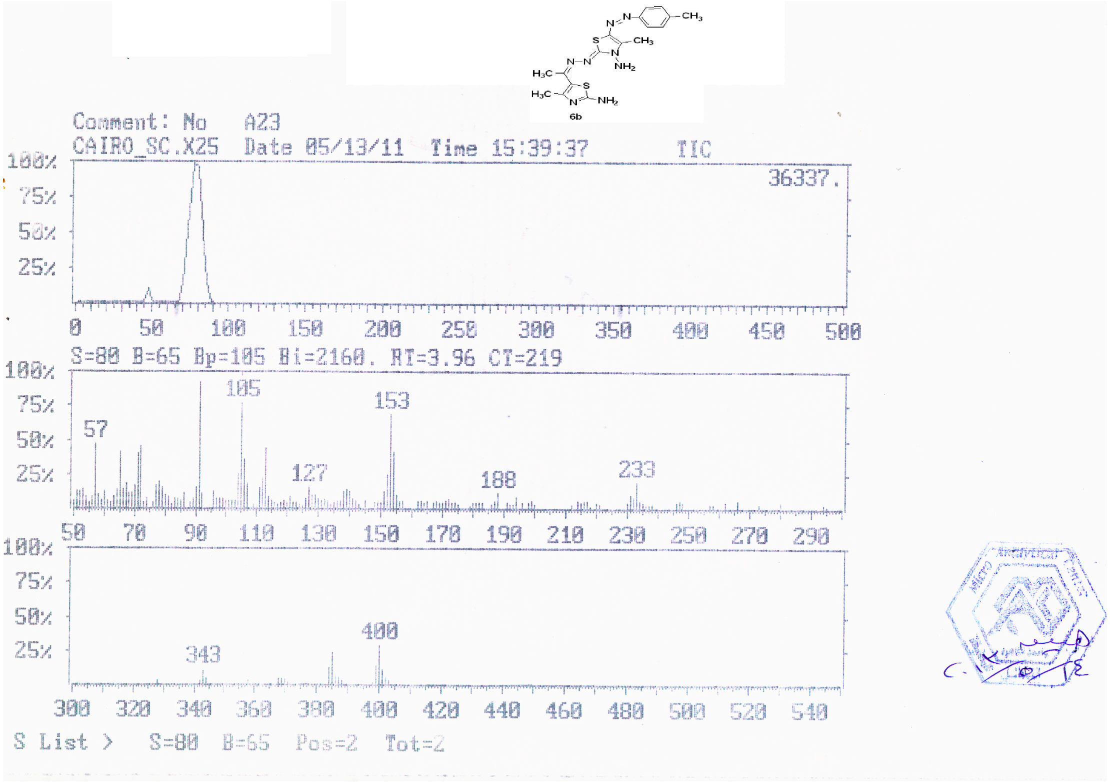

Figure S21. Mass of compound **6b**

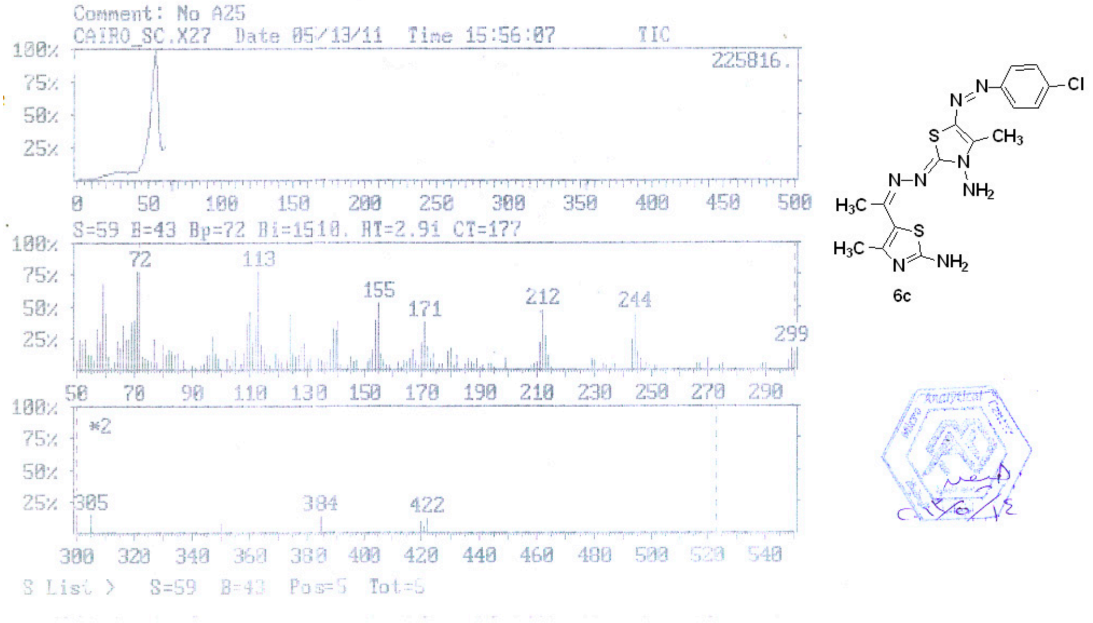

Figure S22. Mass of compound **6c**.
